# Supplementary material for: A Bayesian hierarchical model of trial-to-trial fluctuations in decision criterion
Source: PLoS Comput Biol. 2025 Jul 29;21(7):e1013291. doi: 10.1371/journal.pcbi.1013291 (PMC12367131; doi:10.1371/journal.pcbi.1013291)
Supplement: S1 Appendix — (PDF) [file pcbi.1013291.s001.pdf]

Appendix

Hierarchical Model for Fluctuations in Criterion (hMFC) Details

Notation Let

- N denote the **number of subjects**
- T_i denote the **number of trials** for subject i
- $\eta = (\boldsymbol{\mu}_w, \boldsymbol{\sigma}^2_w, \mu_a, \sigma^2_a, \mu_{\sigma^2}, \beta_{\sigma^2}, \sigma^2_{\mu_x})$ denote the **group-level (global) parameters**.
- $\theta_i = (\boldsymbol{w}_i, a_i, \sigma^2_i, \mu_{x,i})$ denote the **subject-level (local) parameters** for subject i
- $x_{i,t} \in \mathbb{R}$ for $t \in 1, \ldots, T_i$ denote the sequence of **latent states** for subject i.
- $\boldsymbol{u}_{i,t} \in \mathbb{R}^p$ for $t \in 1, \ldots, T_i$ denote the sequence of **inputs** for subject i.
- $y_{i,t} \in \{0, 1\}$ for $t \in 1, \ldots, T_i$ denote the sequence of **binary observations** for subject i.

The Hierarchical Model for Fluctuations in Criterion (hMFC) defines a joint distribution over latent states, observations, and parameters given the inputs,

$$p(\{\{x_{i,t}, y_{i,t}\}_{t=1}^{T_i}, \theta_i\}_{i=1}^{N}, \eta \mid \{\{\boldsymbol{u}_{i,t}\}_{t=1}^{T_i}\}_{i=1}^{N}) = \\ p(\eta) \times \prod_{i=1}^{N} \Bigg(p(\theta_i \mid \eta) \times \left[\mathrm{N}(x_{i,1} \mid \mu_{x,i}, 1) \prod_{t=2}^{T_i} \mathrm{N}(x_{i,t} \mid a_i x_{i,t-1} + (1-a_i)\mu_{x,i}, \sigma^2_i) \right] \\ \times \left[\prod_{t=1}^{T_i} \mathrm{Bern}\big(y_{i,t} \mid f(x_{i,t} + \boldsymbol{w}_i^\top \boldsymbol{u}_{i,t})\big) \right] \Bigg) \quad (1)$$

where $f(z) = 1/(1+e^{-z})$ denotes the logistic function and $(1-a_i)\mu_{x,i}$ is the intercept b_i.

$$p(\theta_i \mid \eta) = \mathrm{N}(\boldsymbol{w}_i \mid \boldsymbol{\mu}_w, \mathrm{diag}(\boldsymbol{\sigma}^2_w)) \times \mathrm{TruncNorm}(a_i \mid \mu_a, \sigma^2_a, [0,1]) \times \mathrm{IGa}(\sigma^2_i \mid \mu_{\sigma^2}, \beta_{\sigma^2}) \times \mathrm{N}(\mu_{x,i} \mid 0, \sigma^2_{\mu_x}) \quad (2)$$

is then the hierarchical prior. The truncated normal distribution ensures that $a_i \in [0,1]$, so that the latent state dynamics for subject i are stable. The hierarchical Gaussian prior on weights $\boldsymbol{w}_i \in \mathbb{R}^p$ allows for sharing of statistical strength across subjects.

We assume weakly informative priors over the global parameters,

$$\begin{aligned} p(\eta) &= \mathrm{Unif}(\mu_a; [0,1]) \times \mathrm{Unif}(\sigma_a; [0, \sigma_a^{(\max)}]) \times \mathrm{N}(\boldsymbol{\mu}_w \mid 0, \boldsymbol{\sigma}_{\boldsymbol{w}}^2 I) \\ &\times \mathrm{IGa}(\boldsymbol{\sigma}_{\boldsymbol{w}}^2 \mid \alpha_{\sigma_w^2}, \beta_{\sigma_w^2}) \times \mathrm{IGa}(\boldsymbol{\sigma}_{\mu_x}^2 \mid \alpha_{\sigma_{\mu_x}^2}, \beta_{\sigma_{\mu_x}^2}) \times \mathrm{Unif}(\mu_{\sigma^2}; [0,10]) \times \mathrm{Unif}(\beta_{\sigma^2}; [0,10]) \end{aligned} \quad (3)$$

with $\boldsymbol{\sigma}_{\boldsymbol{w}}^2 \to \infty$, $\alpha_{\sigma_w^2} \to 0$, $\beta_{\sigma_w^2} \to 0$, $\alpha_{\sigma_{\mu_x}^2} \to 0$, $\beta_{\sigma_{\mu_x}^2} \to 0$. The uniform priors for μ_a, σ_a, μ_{σ^2}, and β_{σ^2} allow for a wide range of global parameters, while still enforcing marginal stability constraints.

We set $\sigma_a^{(\max)} = 0.2$ to penalize the model for using very different values for a_i across subjects. Nevertheless, this value is a generous upper bound.

Posterior Inference

We designed an augmented, blocked Gibbs sampling algorithm to estimate the posterior distribution, $p(\{\{x_{i,t}\}_{t=1}^{T_i}, \theta_i\}_{i=1}^N, \eta \mid \{\{\boldsymbol{u}_{i,t}, y_{i,t}\}_{t=1}^{T_i}\}_{i=1}^N)$.

Gibbs sampling the latent states

The trick is to represent the Bernoulli likelihood as a scale-mean mixture of Gaussians using the Pólya-gamma (PG) augmentation (**?**),

$$\mathrm{Bern}(y \mid \sigma(\psi)) \propto e^{(y-\frac{1}{2})\psi} \int e^{-\frac{1}{2}\omega\psi^2} \, \mathrm{PG}(\omega \mid 1, 0) \, \mathrm{d}\omega, \quad (4)$$

where $\mathrm{PG}(\omega \mid 1, 0)$ denotes the standard Pólya-gamma density on $\omega \in \mathbb{R}_+$. In our case, $\psi_{it} = x_{it} + \boldsymbol{w}_i^\top \boldsymbol{u}_{it}$. After augmenting the model with auxiliary variables ω_{it}, the log likelihood

as a function of x_{it} is,

$$\log p(y_{it} \mid x_{it}, \omega_{it}, \boldsymbol{u}_{it}, \theta_i) = h_{it} x_{it} - \frac{1}{2}\omega_{it} x_{it}^2 \tag{5}$$

where

$$h_{it} = y_{it} - \omega_{it} \boldsymbol{w}_i^\top \boldsymbol{u}_{it} - \frac{1}{2} \tag{6}$$

is the precision-weighted mean, which we recognize as a kind of residual.

After augmentation, the conditional probability of x_i is an exponentiated quadratic,

$$p(x_i \mid y_i, \boldsymbol{u}_i, \omega_i, \theta_i) = \\ \mathrm{N}(x_{i,1} \mid \mu_{x,i}, 1) \prod_{t=2}^{T_i} \mathrm{N}(x_{i,t} \mid a_i x_{i,t-1} + (1 - a_i)\mu_{x,i}, \sigma_i^2) \prod_{t=1}^{T} \exp\left\{ h_{it} x_{it} - \frac{1}{2}\omega_{it} x_{it}^2 \right\}. \tag{7}$$

We recognize this as a chain-structured Gaussian graphical model — i.e., a linear Gaussian dynamical system — over x_i. We use information-form message passing algorithms implemented in Dynamax (**?**) to sample the latent states from their conditional distribution given the emissions, inputs, parameters, and auxiliary PG variables.

Sampling the Pólya-gamma augmentation variables

The conditional distribution over auxiliary variables ω_{it} given the states and parameters is,

$$p(\omega_{it} \mid y_{it}, x_{it}, \boldsymbol{u}_{it}, \theta_i) = \mathrm{PG}(\omega_{it} \mid 1, \psi_{it}) \tag{8}$$
$$\psi_{it} = x_{it} + \boldsymbol{w}_i^\top \boldsymbol{u}_{it}$$

There are highly efficient rejection sampling algorithms for the $\mathrm{PG}(1, \psi)$ distribution (**?**), but we can also use a naïve sampling algorithm based on a representation of the PG distribution as a weighted sum of gamma random variates (**?**).

Note that the augmentation variables (one per subject and trial) are conditionally independent of one another and thus can be sampled in parallel. Our implementation using the JAX library (**?**) takes advantage of this opportunity for fast parallel sampling on GPUs.

Sampling the per-subject (local) parameters

Input weights After augmentation, log likelihood as a function of the weights $\boldsymbol{w}_i$ for subject i is,

$$\log p(y_{it} \mid x_{it}, \omega_{it}, \boldsymbol{u}_{it}, \theta_i) = \boldsymbol{h}_{it}^\top \boldsymbol{w}_i - \frac{1}{2}\boldsymbol{w}_i^\top(\omega_{it}\boldsymbol{u}_i\boldsymbol{u}_i^\top)\boldsymbol{w}_i + c \tag{9}$$

where $\boldsymbol{h}_{it} = (y_{it} - \omega_{it}x_{it} - \frac{1}{2})\boldsymbol{u}_i$ and c is constant w.r.t. $\boldsymbol{w}_i$.

Again, this quadratic log probability is conditionally conjugate with the Gaussian prior on the weights, $\boldsymbol{w}_i \sim \mathrm{N}(\boldsymbol{\mu}_w, \mathrm{diag}(\boldsymbol{\sigma}_w^2))$. The conditional posterior distribution is,

$$\begin{aligned} p(\boldsymbol{w}_i \mid \{y_{it}, x_{it}, \boldsymbol{u}_{it}\}_{t=1}^{T_i}, \theta_i, \eta) &= \mathrm{N}(\boldsymbol{w}_i \mid \boldsymbol{J}^{-1}\boldsymbol{h}, \boldsymbol{J}^{-1}) \\ \boldsymbol{J} &= \mathrm{diag}(\boldsymbol{\sigma}_w^{-2}) + \sum_{t=1}^{T_i} \omega_{it}\boldsymbol{u}_i\boldsymbol{u}_i^\top \\ \boldsymbol{h} &= \mathrm{diag}(\boldsymbol{\sigma}_w^{-2})\boldsymbol{\mu}_w + \sum_{t=1}^{T_i}\left(y_{it} - \omega_{it}x_{it} - \frac{1}{2}\right)\boldsymbol{u}_i \end{aligned} \tag{10}$$

Dynamics coefficient The truncated normal prior on a_i is conjugate with the linear Gaussian dynamics model. Given the latent states,

$$p(a_i \mid x_i, \eta) \propto \mathrm{TruncNorm}(a_i \mid \mu_a, \sigma_a^2)\prod_{t=2}^{T_i}\mathrm{N}(x_{i,t} \mid a_i x_{i,t-1} + (1-a_i)\mu_{x,i}, \sigma_i^2) \tag{11}$$

$$\propto \exp\left\{-\frac{1}{2\sigma_a^2}(a_i - \mu_{x,i})^2 - \frac{1}{2\sigma_i^2}\sum_{t=2}^{T_i}\left(\Delta_{i,t} - a_i\Delta_{i,t-1}\right)^2\right\}\mathbb{I}[a_i \in [0,1]] \tag{12}$$

where $\Delta_{i,t} = (x_{i,t} - \mu_{x,i})$. Simplifying this expression yields,

$$
\begin{aligned}
p(a_i \mid x_i, \eta) &= \text{TruncNorm}(a_i \mid J^{-1}h, J^{-1}; [0,1]) \\
J &= \sigma_a^{-2} + \sigma_i^{-2} \sum_{t=2}^{T_i} \Delta_{i,t-1}^2 \\
h &= \sigma_a^{-2}\mu_a + \sigma_i^{-2} \sum_{t=2}^{T_i} \Delta_{i,t}\Delta_{i,t-1}
\end{aligned}
\tag{13}
$$

This is a truncated normal distribution with mean $J^{-1}h$ and variance J^{-1}, constrained to $a_i \in [0,1]$.

Dynamics noise variance The inverse gamma prior is conjugate with the Gaussian noise model. The conditional posterior distribution is,

$$
\begin{aligned}
p(\sigma_i^2 \mid x_i, a_i, \eta) &= \text{IGa}(\alpha', \beta') \\
\alpha' &= \alpha + \frac{T_i - 1}{2} \\
\beta' &= \beta + \frac{1}{2} \sum_{t=2}^{T_i} (x_{i,t} - a_i x_{i,t-1} - (1 - a_i)\mu_{x,i})^2
\end{aligned}
\tag{14}
$$

Per-subject bias The per-subject bias, $\mu_{x,i}$, is conjugate with a Gaussian prior. Given the latent states and dynamics parameters,

$$
p(\mu_{x,i} \mid x_i, a_i, \sigma_{\mu_x}^2) \propto \text{N}(\mu_{x,i} \mid 0, 1)\,\text{N}(x_{i,1} \mid \mu_{x,i}, 1) \prod_{t=2}^{T_i} \text{N}(x_{i,t} \mid a_i x_{i,t-1} + (1 - a_i)\mu_{x,i}, \sigma_i^2) \tag{15}
$$

$$
= \text{N}(\mu_{x,i} \mid J^{-1}h, J^{-1}) \tag{16}
$$

where

$$J = \frac{1}{\sigma^2_{\mu_x}} + 1 + \sum_{t=2}^{T_i} \frac{(1-a_i)^2}{\sigma_i^2}$$
$$h = x_{i,1} + \sum_{t=2}^{T_i} \frac{(x_{i,t} - a_i x_{i,t-1})(1-a_i)}{\sigma_i^2}$$

Sampling the group-level (global) parameters

The global parameters $\boldsymbol{\mu}_w$ and $\boldsymbol{\sigma}^2_w$ admit closed form Gibbs updates. Under their uninformative priors,

$$p(\mu_{w,j} \mid \{w_{i,j}\}_{i=1}^N, \sigma^2_{w,j}) = \mathrm{N}\left(\mu_{w,j} \,\middle|\, \frac{1}{N}\sum_{i=1}^N w_{i,j}, \frac{\sigma^2_{w,j}}{N}\right) \tag{17}$$

and

$$p(\sigma^2_{w,j} \mid \{w_{i,j}\}_{i=1}^N) = \mathrm{IGa}\left(\sigma^2_{w,j} \,\middle|\, \frac{N}{2}, \frac{1}{2}\sum_{i=1}^N (w_{i,j} - \mu_{w,j})^2\right) \tag{18}$$

for $j = 1, \ldots, p$ with p the total number of covariates. Finally, we update the global parameters μ_a, σ^2_a, μ_{σ^2}, and β_{σ^2} using random-walk Metropolis-Hastings with symmetric Gaussian proposal distributions. The parameter α_{σ^2} is then derived as $\beta_{\sigma^2}/\mu_{\sigma^2} + 1$.

Initialization

We initialize the global parameters conservatively, setting $\mu_a = 0.90$, $\sigma_a = 0.1$, $\boldsymbol{\mu}_w = (0, \ldots, 0)^\top$, $\sigma_{w,j} = 1.25$ for all j, $\mu_{\sigma^2} = 0.3$, $\beta_{\sigma^2} = 0.6$, and $\sigma_{\mu_x} = 0.6$. Thus, at initialization, the global prior is biased toward producing per-subject latent states that are reasonably autocorrelated. Then we sample the parameters and states from their conditional distributions given the global prior, without conditioning on the data.

More informative initializations are possible. For example, one could fit a logistic regression

Algorithm 1: Gibbs sampling algorithm for the hierarchical Bernoulli LDS

Input: Observed inputs and choice sequences for N subjects, $\{u_i, y_i\}_{i=1}^{N}$

Initialize the latent states and parameters as described above.

repeat S times to draw S samples

1. Sample the PG augmentation variables $\omega_{i,t}$ according to eq. (8).
2. Sample the local parameters θ_i according to eq. (10-14).
3. Sample latent states x_i using message passing for eq. (7).
4. Sample the global parameters $\boldsymbol{\mu}_w$ and $\boldsymbol{\sigma}^2_w$ according to eq. (17-18).
5. Sample the global parameters μ_a, σ^2_a, α, and β using random-walk Metropolis-Hastings.

end

(without time-varying latent states) for each subject to obtain initial weights $\boldsymbol{w}_i$, and then initialize the per-subject latent states to $x_i = (0, \ldots, 0)^\top$. Based on the initial weights, we can estimate the global weight mean $\boldsymbol{\mu}_w$ and variance $\boldsymbol{\sigma}^2_w$. Combined with the initializations for μ_a, σ^2_a, μ_{σ^2}, $\beta\sigma^2$, and σ_{μ_x} above, this procedure should effectively start the Gibbs sampler near a reasonable basin of the posterior.

Full algorithm

The complete algorithm is given by Algorithm 1.
