# Supplementary figures and images for: A Bayesian hierarchical model of trial-to-trial fluctuations in decision criterion
Source: PLoS Comput Biol. 2025 Jul 29;21(7):e1013291. doi: 10.1371/journal.pcbi.1013291 (PMC12367131; doi:10.1371/journal.pcbi.1013291)

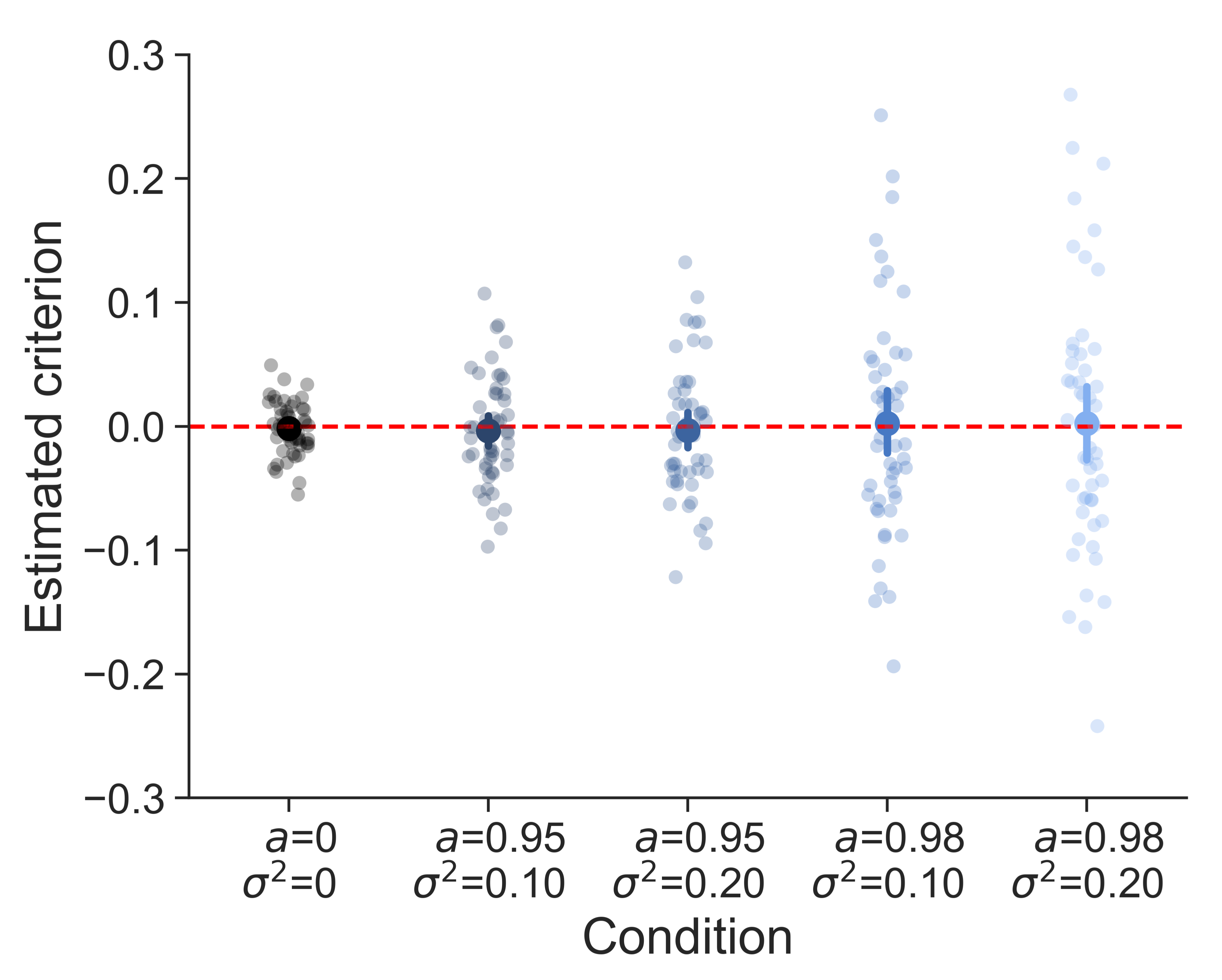

Supplement: S1 Fig — (TIF) [file pcbi.1013291.s002.tif]

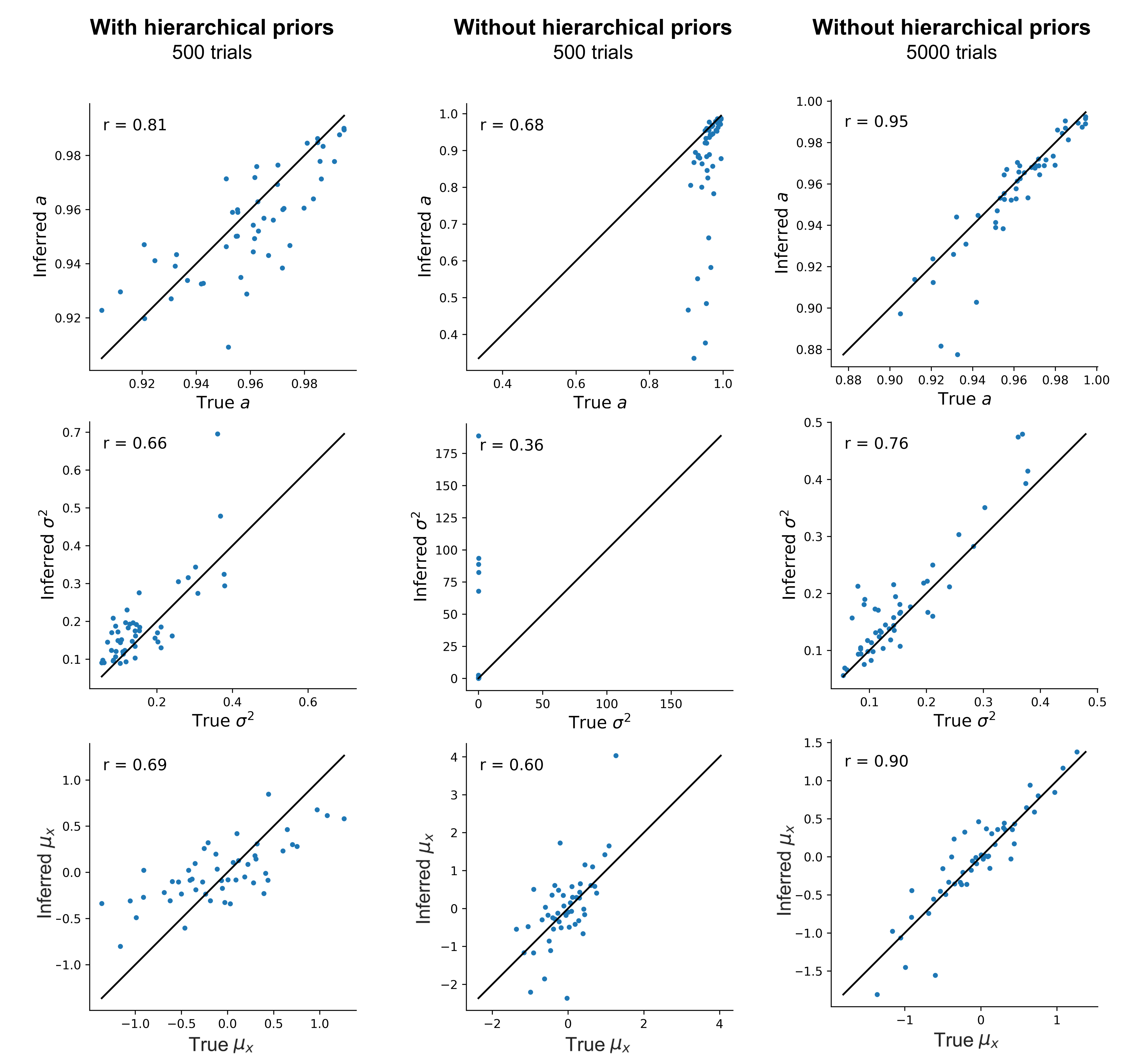

Supplement: S2 Fig — (TIF) [file pcbi.1013291.s003.tif]

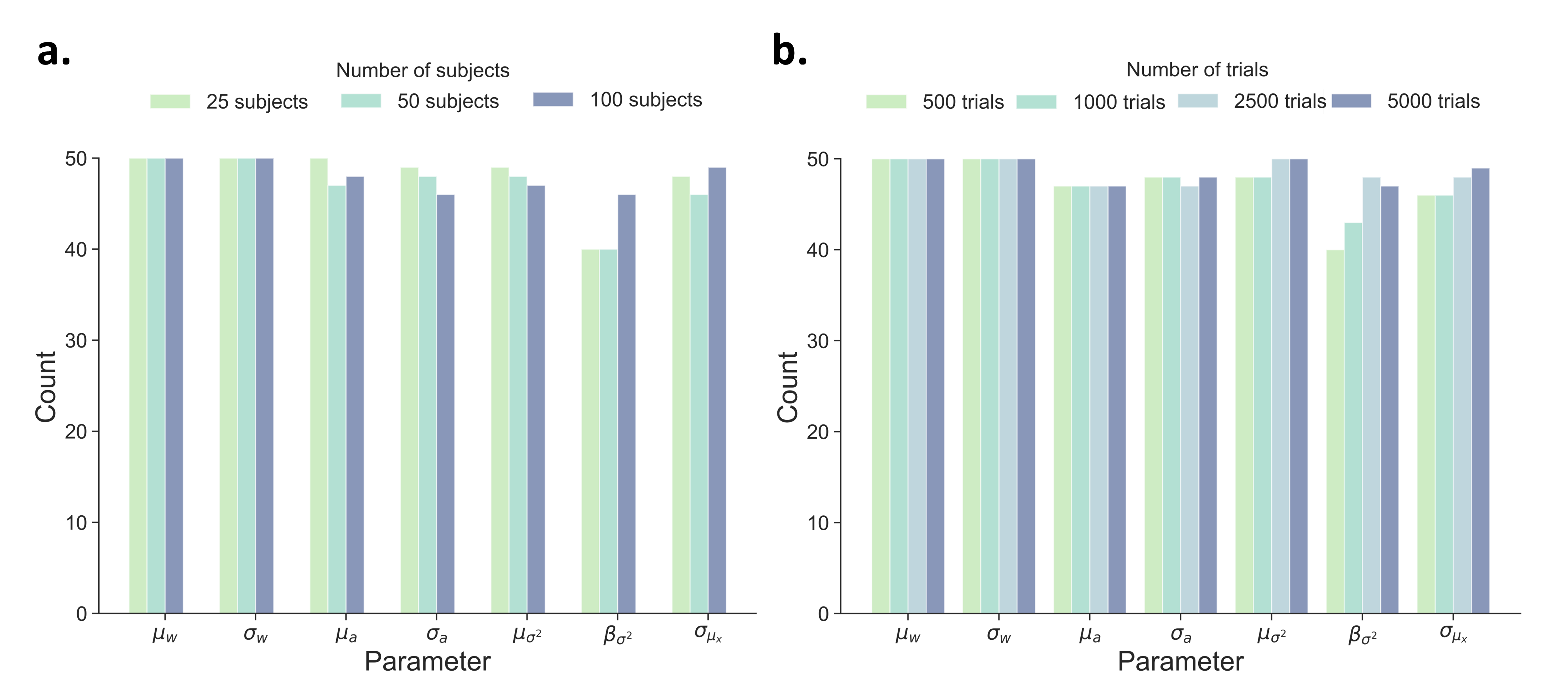

Supplement: S3 Fig — (TIF) [file pcbi.1013291.s004.tif]

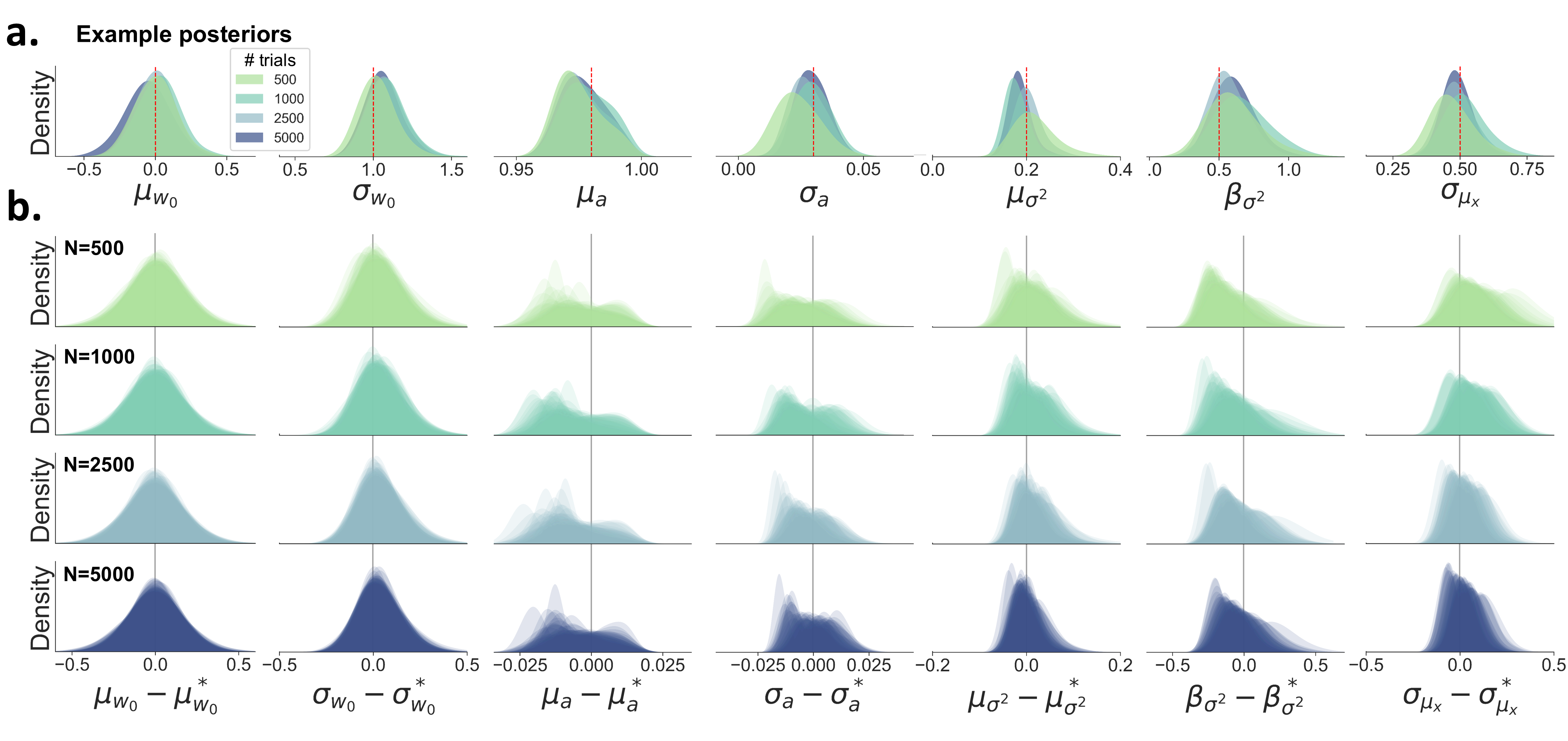

Supplement: S4 Fig — Example posteriors and overlaid posterior distributions are shown. (TIF) [file pcbi.1013291.s005.tif]

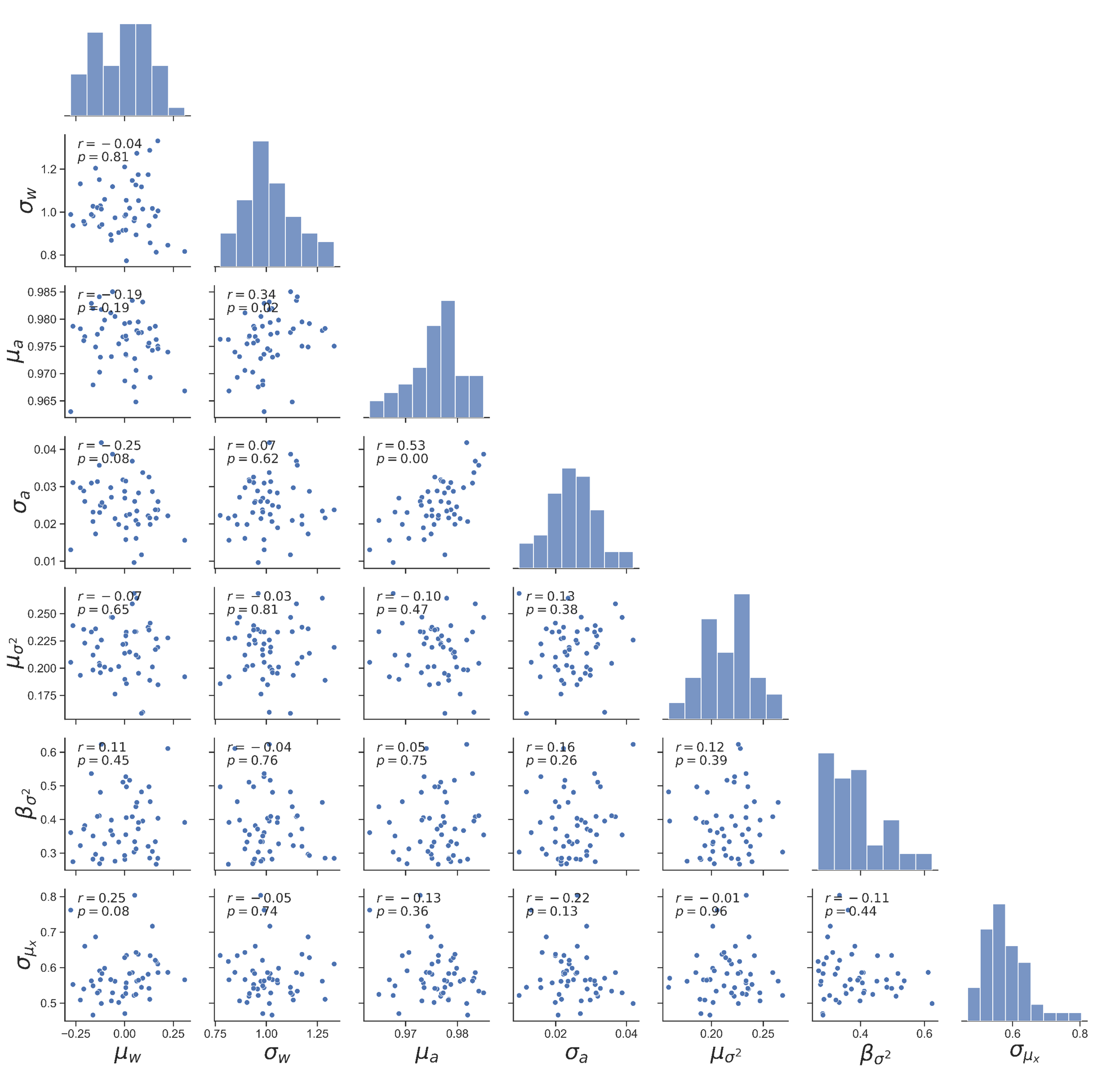

Supplement: S5 Fig — (TIF) [file pcbi.1013291.s006.tif]

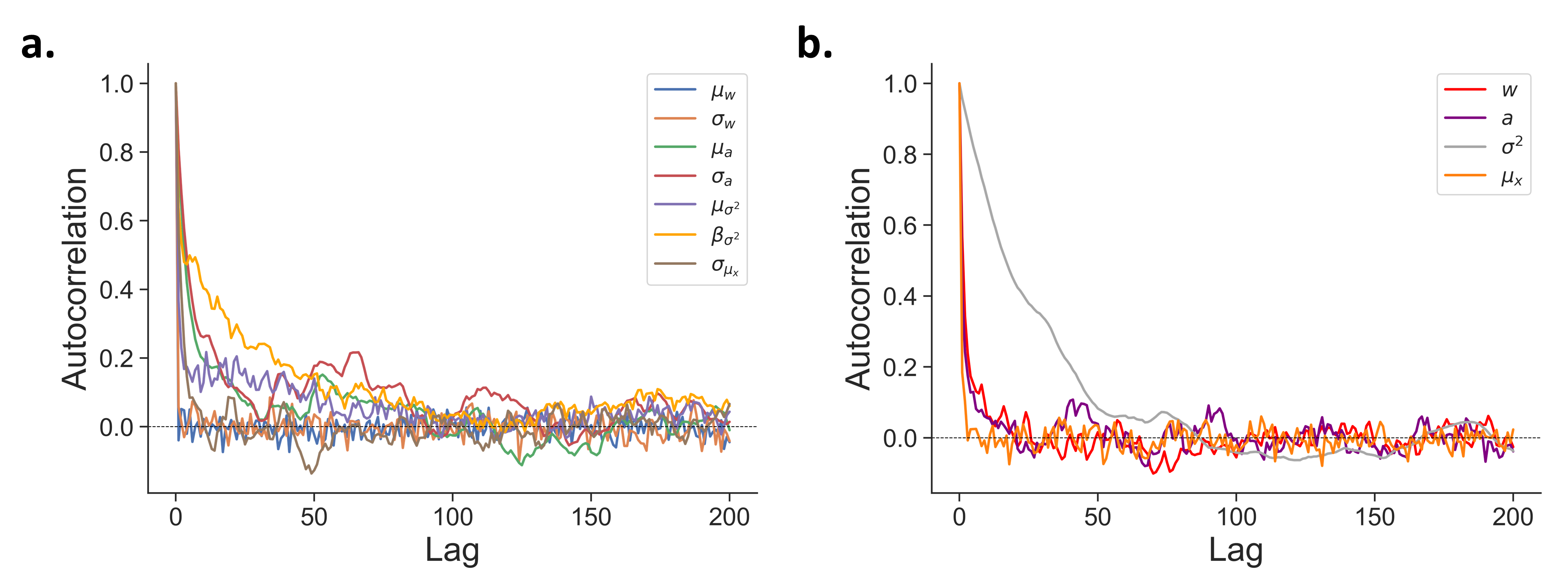

Supplement: S6 Fig — (TIF) [file pcbi.1013291.s007.tif]

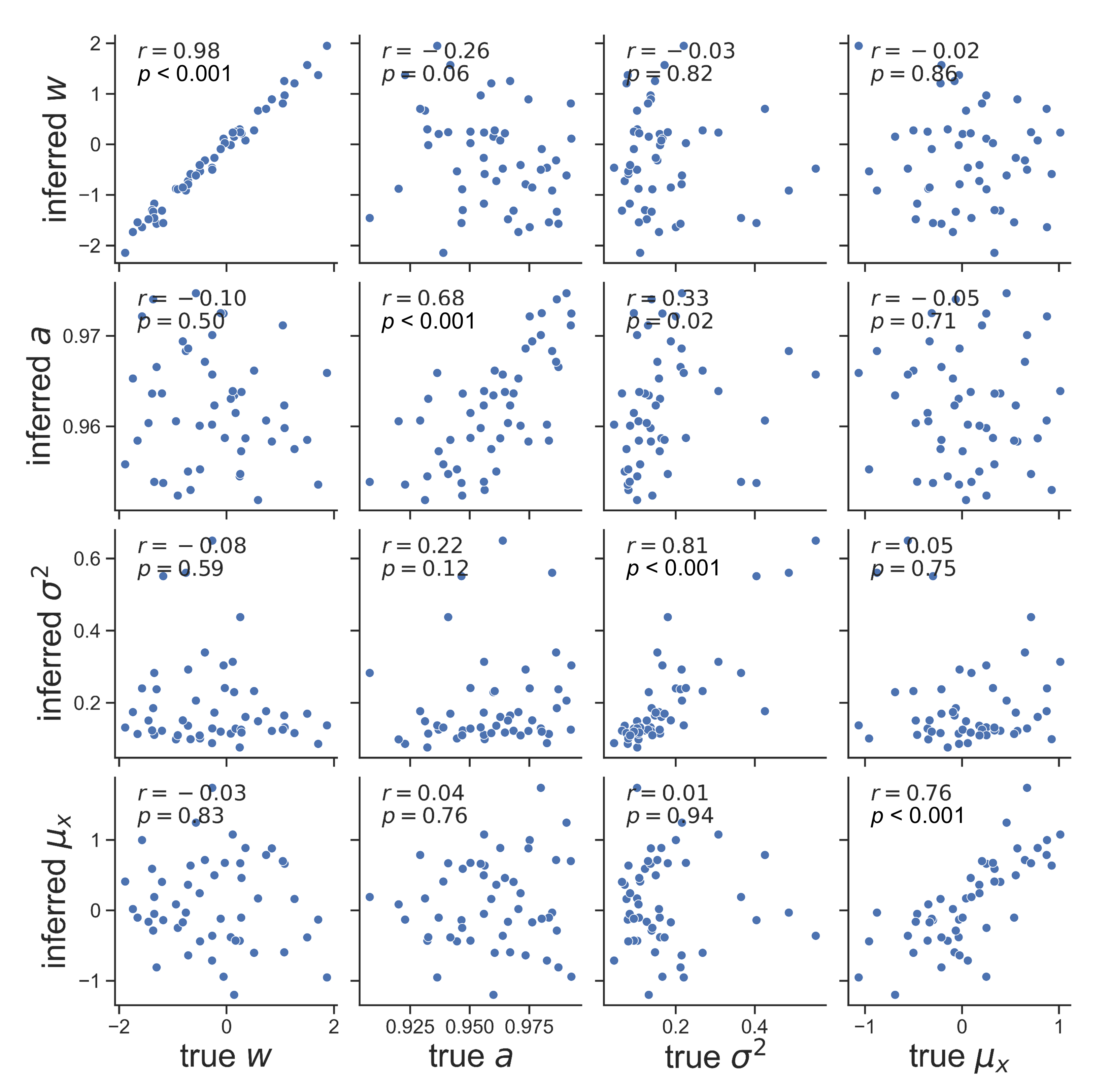

Supplement: S7 Fig — (TIF) [file pcbi.1013291.s008.tif]

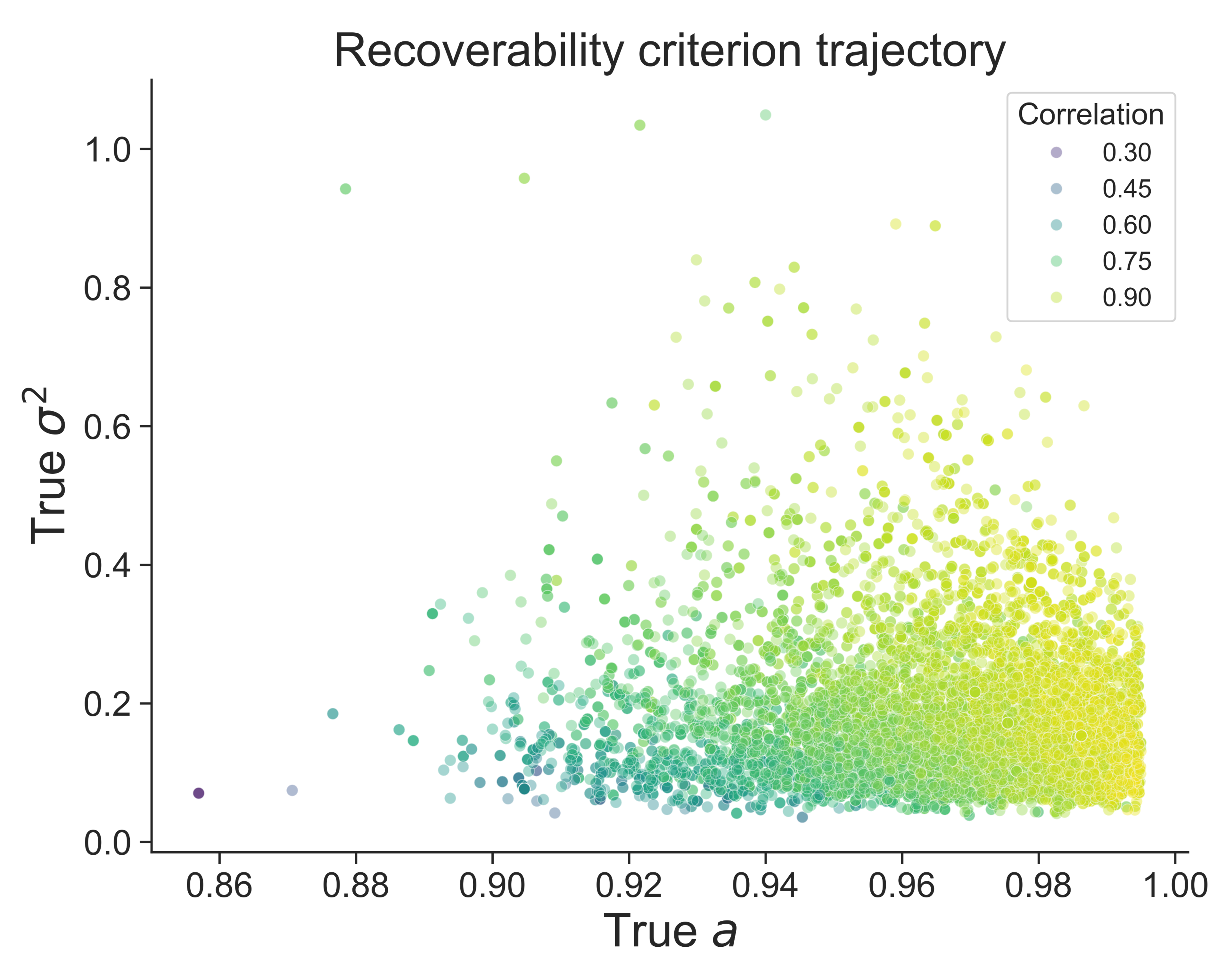

Supplement: S8 Fig — (TIF) [file pcbi.1013291.s009.tif]

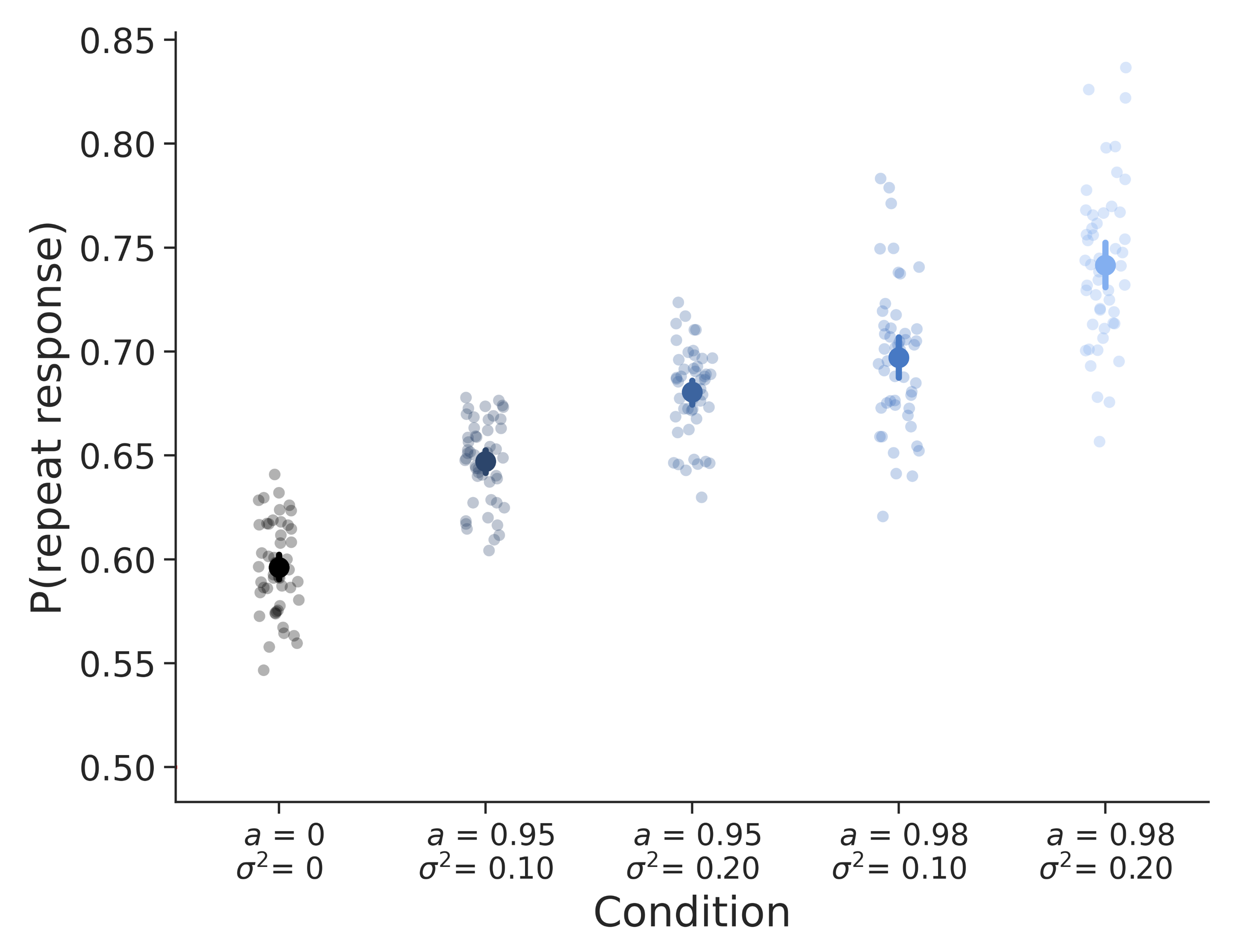

Supplement: S9 Fig — (TIF) [file pcbi.1013291.s010.tif]
