# Supplementary material for: A Bayesian hierarchical model of trial-to-trial fluctuations in decision criterion
Source: PLoS Comput Biol. 2025 Jul 29;21(7):e1013291. doi: 10.1371/journal.pcbi.1013291 (PMC12367131; doi:10.1371/journal.pcbi.1013291)
Supplement: Attachment — Submitted filename: hMFC_reply_reviewers.pdf [file pcbi.1013291.s011.pdf]

Dear Editor,

We thank you and the three Reviewers for providing us with insightful comments and feedback on our manuscript (PCOMPBIOL-D-24-01687). In the current revision, we have carefully addressed all the points raised by the reviewers. Most prominently, we have focused on showing the advantages of the model over previous literature using both empirical and simulated data. In addition, we made two reparameterization changes to the model that fixed slight biases in our original approach. These were discovered thanks to the comments of the Reviewers, and fixing these issues led to an improved parameter recovery. Lastly, we have extended the discussion and now discuss some of the limitations of the model that readers should be aware of.

Please find below a point-by-point reply to each of the comments.

Sincerely,

Robin Vloeberghs, on behalf of all co-authors.

Reviewer #1

In this manuscript, Vloeberghs et al. describe a hierarchical model that tracks trial-to-trial fluctuations in decision criterion. The paper's contributions include code showing how their model can be applied by other researchers. The paper tackles an interesting and relevant problem, and their methods will be of interest to many researchers. However a number of the paper's main claims about the novelty/usefulness of the authors' methods warrant more evidence and/or more precise wording, especially in light of past work. Additionally, the provided demos and discussion warrant some restructuring for the sake of readability and usefulness. I have detailed these points below:

R1.1 Accuracy of estimating fluctuations with lower data requirements

R1.1.1 One of the key claims about the novelty and benefits of the authors' methods, is that it has lower data requirements than previous methods, as it accurately recovers parameter and criterion estimates with as little as 500 trials. However, this claim needs to be qualified for the following reasons:

In figure 3, we see that estimated parameters of the criterion drift (mu_a, sigma_a, alpha and beta) show a **systematic bias** - they are all systematically underestimated even with increasing numbers of subjects - the authors do not address this bias in recovery.

We thank the reviewer for these comments. Indeed, in the previous version of our manuscript the recovery of the group-level parameter showed a systematic bias. We originally attributed this bias to peculiarities of our simulations, but based on your prompt, we investigated it more thoroughly. First, we tested whether our Gibbs steps were mathematically correct using the Geweke test (Geweke, 2004, Journal of the American Statistical Association). The test revealed that our estimation procedure was indeed not entirely bias-free, a problem we traced back to the mean-centering of the criterion trajectories. As a reminder, this mean-centering was needed to avoid a

trade-off between the intercept in the Bernoulli probability ($w_{i,0}$) and the overall mean of the criterion trajectories, as both can capture an overall bias. In the revised version of our model, we now circumvent the need of mean-centering by implementing an intercept in the AR(1) process (b_i), and removing the intercept in the Bernoulli probability. The intercept b_i is estimated indirectly as $(1 - a_i)\ \mu_{x,i}$, following from the formula of the AR(1) mean. Assuming a Gaussian prior we then estimate $\mu_{x,i}$ with the Gibbs sampler. Further investigation using the Geweke test confirmed that this solved the issue. In addition, thanks to a suggestion from reviewer 2 (R2.1), we discovered that the estimates for α_{σ^2} and β_{σ^2}, the parameters of inverse gamma (IG) prior for σ_i^2, were highly correlated (r = .95), making it challenging to recover these parameters. To reduce this correlation, we reparametrized this prior in terms of the mean μ_{σ^2} instead of the shape α_{σ^2}. Then, α_{σ^2} is equivalent to $\beta_{\sigma^2}/\mu_{\sigma^2}$ + 1, which follows from the mean of an IG distribution.

After implementing all the changes outlined above, we re-ran the parameter recovery with 500 trials per subject (i.e. simulated 50 datasets with a varying number of subject (25, 50, 100) per dataset). Despite the changes in the model and the passing Geweke tests, we still see a slight underestimation for μ_a, σ_a, and β_{σ^2} (Figure 3). After thorough investigation, we suspect that the bias is due to the true μ_a being so close to the upper boundary of 1, which causes the posterior distribution of this parameter to be asymmetric and leads to compensatory effects for the other parameter estimates.

In the paper on p. 18 and inserted below we have updated Figure 3 and its caption and now explicitly mention the underestimation and what might possibly drive it.

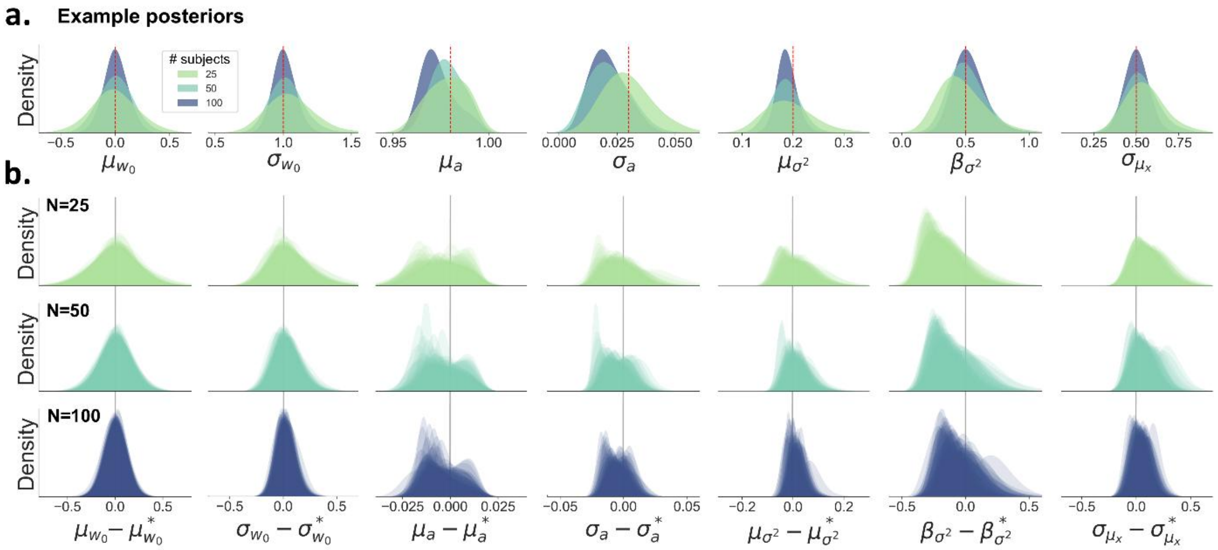

Figure 3: An overview of the estimated posteriors distributions for the group-level (global) parameters when varying the number of subjects per dataset, with 500 trials per subject. A) The posterior distributions become more narrow as the number of subjects increases. The true parameter values are indicated by the red dashed line. B) Each row shows the overlaid posteriors for all 50 simulated datasets with a varying number of subjects per dataset. The estimated posteriors are corrected and centered on the true value (denoted by an asterisk). For μ_w and σ_w the true value is defined as the mean or standard deviation of the true per-subject parameters w_i within each dataset. Due to boundaries in the parameter space, with σ_w, σ_a, μ_{σ^2}, and β_{σ^2} being strictly positive, the posterior distributions can be skewed. It should be noted that there is a slight

underestimation for μ_a, σ_a, and β_{σ^2}. This is presumably due to the true μ_a being so close to the upper boundary of 1, which causes the posterior distribution of this parameter to be asymmetric and possibly leading to compensatory effects for the other parameter estimates. Overall, we see an excellent recovery of the group-level (global) parameters.

R1.1.2 The authors claim that recovered **estimate lies in the 95% credible intervals but these intervals are not shown**.

The Reviewer is correct that the credible intervals (CI) were not shown in the previous version of the manuscript. The number of times (out of the 50 simulated datasets) the true parameter value falls in the 95% CI is now added as a supplementary figure (Figure S3, copied below). In panel A the number of subjects (500 trials per subject) was varied over datasets, whereas in panel B each dataset consisted of 50 subject but varied in the number of trials per subject. For most parameters the true value falls in the 95% CI in almost all cases. Most importantly, for μ_w and σ_w this is the case for 50/50 datasets. Although β_{σ^2} is harder to recover with a lower number of trials or subjects (40/50 datasets), its recovery improves with more trials or subjects. We now refer to Figure S3 in the Manuscript on p. 17, lines 361-364:

"For at least 46/50 datasets, the generative value lies within the 95% credible interval of the posterior estimates (Fig. S3). Only β_{σ^2} seemed harder to recover (40/50 datasets) at lower trial counts, but this did not impact the recovery of the criterion trajectories (Fig. 5A) and recovery of the parameter improved with higher trial numbers (48/50 datasets). Most important, for μ_w and σ_w the 95% credible interval contains the true value for 50/50 datasets."

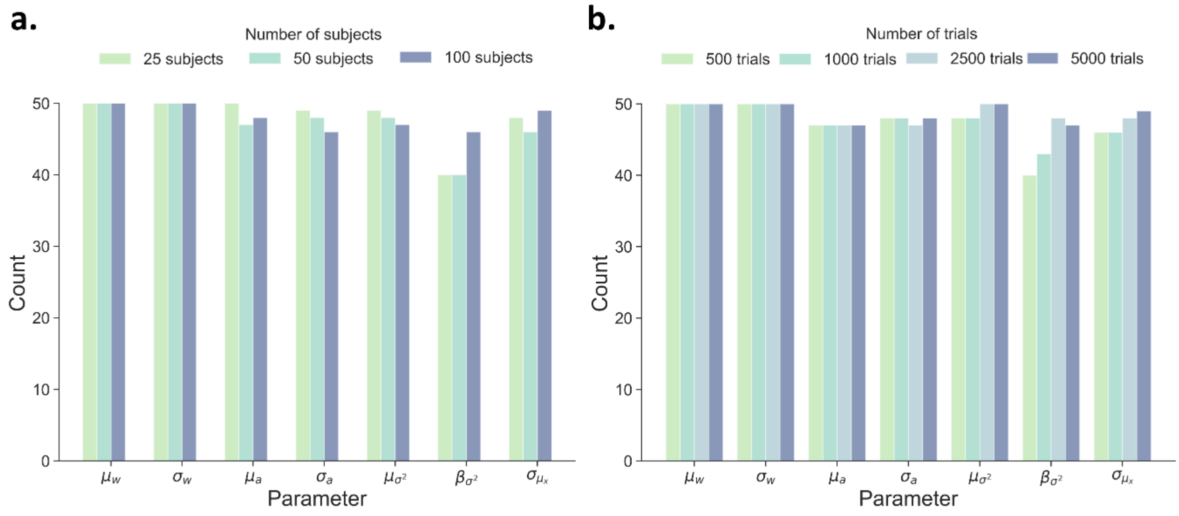

Supplementary Figure S3: The number of times the true parameter value falls in the 95% credible interval out of 50 datasets in function of number of subjects (A), and number of trials (B).

R1.1.3 In figure 4a, we see that the spearman correlation of recovered parameters drops off significantly (close to 60%) for some of the parameters when trial counts reach 500 trials. Relatedly, both Roy et al and Gupta & Brody do in fact fit datasets on the order of 1000 trials (Fig 7 in Roy et al, Fig 3E of Gupta & Brody) showing similar levels of recovery at such low trial counts. Due to these issues, it is unclear what performance advantage the hMFC is buying at these low trial counts compared to previous techniques - perhaps the authors **could show the advantage**

of fitting with v.s. without hierarchical priors to show what the hierarchical nature of the model adds.

The Reviewer is correct that previous work has indeed fitted models to datasets with 1000 trials. However, these models did not explicitly estimate the autoregressive coefficient. As we now show in Supplementary Figure S1 (copied below), estimating this parameter at low trial counts in a non-hierarchical model is not feasible. To demonstrate this, we simulated 5 datasets consisting of 50 subjects with 500 trials each and fitted hMFC once with and once without hierarchical priors. For the latter, we simply set the prior variances to large values without updating them, thereby making the priors uninformative. The plot below shows one representative dataset. Fitting hMFC with hierarchical implementation results in excellent recovery of a_i *and* σ_i^2*. However, when removing the hierarchical priors, we see that the recovery for that same dataset becomes quite poor. To compensate for an underestimation of* a_i *the models overestimates* σ_i^2*. Also, the recovery of* μ_x *suffers. Importantly, this poor recovery without a hierarchical structure is directly related to the lack of datapoints. Indeed, when running the non-hierarchical model with N=5000 trials per subject the model is able to recover the parameters well despite the absence of hierarchical priors. Altogether, this finding indicates that the presence of hierarchical priors benefits the model for the parameter recovery of the AR(1) model. We now refer to Figure S1 in the Manuscript on p. 10, lines 231-237:*

> *"Second, instead of fixing* a_i *in the AR(1) to .9995 (Gupta & Brody, 2022) or 1 (Roy et al., 2021) we implement this in our model as a free parameter. This is of crucial importance because when the true value for* a_i *deviates from the assumed value, it causes biases in the parameters estimates and a poorer recovery of the criterion fluctuations, as will be illustrated in fig.7 using empirical and simulating data. Having* a_i *as a free parameter does require a hierarchical estimation approach for good recovery at lower trial numbers (fig. S1)."*

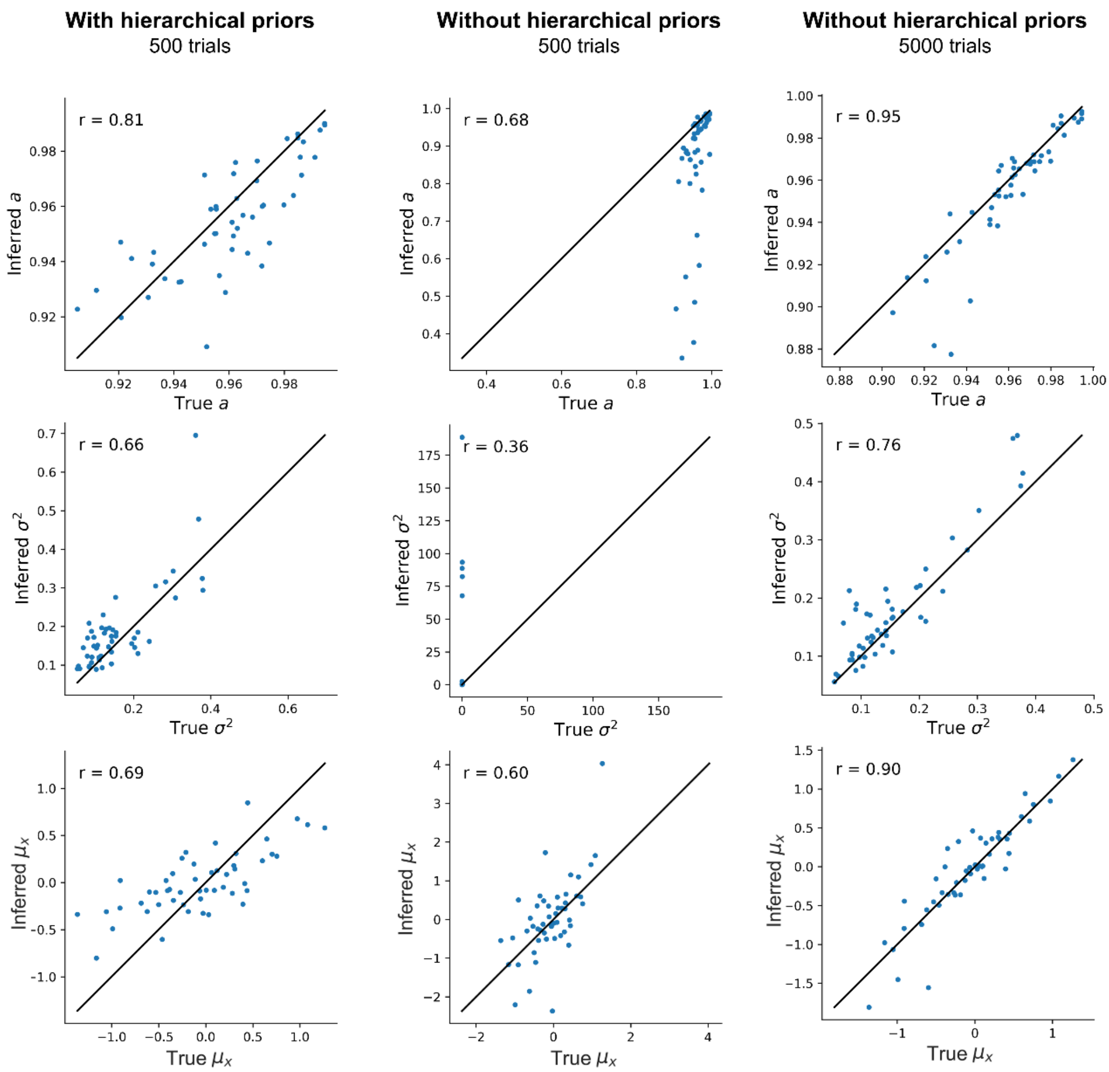

Figure S1: The necessity of the hierarchical structure with low trial counts. *Recovery of per-subject parameters of the AR(1) parameters for a representative dataset (50 subjects). When hMFC is fitted with hierarchical priors the parameters can be well recovered. However, without hierarchical priors the parameters are harder to recover. With more trials, the recovery improves, despite the absence of the hierarchical priors.*

R1.2 Benefits of estimating rather than fixing certain parameters

The authors note that in their model, they treat the autoregressive coefficient "a" as a free parameter, rather than fixing it at 1 or 0.9995 as Roy et al and Gupta & Brody have done. However it is unclear how beneficial this actually is:

In fig 4b, **one can see that very few different values of "a" are actually tested, within a narrow range of 0.94-1**. This is probably owing to the fact that as shown in Supplementary Figure S3, the correlation between true and estimated trajectory drops off quickly as "a" values decrease below 0.99. As mentioned before, recovery of "a" values at 500 trials is quite poor (~60%) however, from Fig 5a the recovery of the trajectory doesn't suffer due to this misestimation. This is likely because the process of smoothing to estimate latent trajectories is tolerant to slight mis-specification of "a", hence it is **unclear how much value we are getting from estimating it as a free parameter**.

To show the benefits of estimating a_i as a free parameter, we now showcase the results of fitting hMFC to the empirical dataset of Shekhar & Rahnev (2021) consisting of 20 subjects with 2970 trials each. Note that fitting an empirical datasets was also requested by reviewer 3 (R3.2). After fitting hMFC to this dataset, we simulated responses and criterion fluctuations with the estimated empirical per-subject parameter values. To investigate what the added value is of a_i being a free parameter, we fitted two versions of hMFC: one where a_i is freely estimated, and the other where a_i is fixed to .9995, as in Gupta and Brody (2022). First, when freely estimating a_i we see an excellent recovery of a_i, and σ_i^2 (Figure 7). In contrast, when a_i is fixed we see that the recovery of σ_i^2 suffers. By underestimating σ_i^2 and keeping the error variance small the model tries to compensate for a_i being systematically too high (i.e., fixed to .9995). Most importantly, the impact on the estimation of the criterion trajectory is shown for an example subject (only first 500 trials shown). The estimation of a_i allows the model to capture the fine-grained dynamics of the trajectory. In contrast, with a_i fixed it can only capture a very general pattern. Across all subjects, the correlation between the true and estimated criterion trajectory is significantly higher ($t = 7.37$, $p < .001$) when a_i is estimated compared to fixed (Figure R1.2B). Also, the root mean squared error (RMSE) is significantly lower ($t = -8.30$, $p < .001$) with a_i as a free parameter. Additionally, these simulations indicate that empirical values for a_i can go much lower than .9995 or 1, and that hMFC can successfully recover these values. In sum, the simulations based on the empirical estimates of Shekhar and Rahnev (2021) show that assuming a fixed value for a_i results in poorer model fits when the actual values deviate from the assumed value. However, as demonstrated in R1.1, treating a_i as a free parameter requires hierarchical priors for a proper recovery at lower trial counts. We now refer to this figure in the Manuscript on p. 10, lines 231-237:

> *"Second, instead of fixing a_i in the AR(1) to .9995 (Gupta & Brody, 2022) or 1 (Roy et al., 2021) we implement this in our model as a free parameter. This is of crucial importance because when the true value for a_i deviates from the assumed value, it causes biases in the parameter estimates and a poorer recovery of the criterion fluctuations, as illustrated in fig.7 using empirical and simulated data. Having a_i as a free parameter does require a hierarchical estimation approach for good recovery at lower trial numbers (fig. S1)."*

In addition we have added a paragraph "Applying hMFC to an empirical dataset" in the Results section on p. 23, lines 433-449:

> *"In the previous sections we have shown that hMFC accurately recovers the generative parameters on simulated data, and that it corrects for biases that occur when not measuring criterion fluctuations. As a final endeavor, we effectively fitted hMFC to an empirical dataset by Shekhar and Rahnev (2021), consisting of 20 subjects with 2790 trials each. In addition, using these empirical fits we will demonstrate the benefit of having the autoregressive coefficient a_i as a free parameter, instead of assuming a fixed value (Gupta and Brody, 2022; Roy et al., 2021) by simulating data based on the empirical parameter estimates.*
>
> *Estimating hMFC to the empirical dataset reveals values ranging from 0.81 to 0.96 for a_i, and values between 0.05 and 0.35 for σ_i^2 (fig. 7A). Using the per-subject parameter estimates and the empirical data we simulated a new dataset and fitted two versions of hMFC in which a_i is freely estimated or in which a_i is fixed to .9995. As can be seen in fig.*

7B-C hMFC is able to recover the parameter well when a_i is freely estimated. However, when a_i is fixed both the recovery of the parameters and the criterion trajectories suffer. In fact, fig. 7D shows that when a_i is fixed the correlation between the true and inferred criterion trajectory is consistently lower ($t = 7.37$, $p < 0.001$) and the root mean square error (RMSE) higher ($t = -8.30$, $p < 0.001$). These results show that hMFC is able to recover the parameter range found in empirical data, and demonstrate the importance of having a_i as a free parameter."

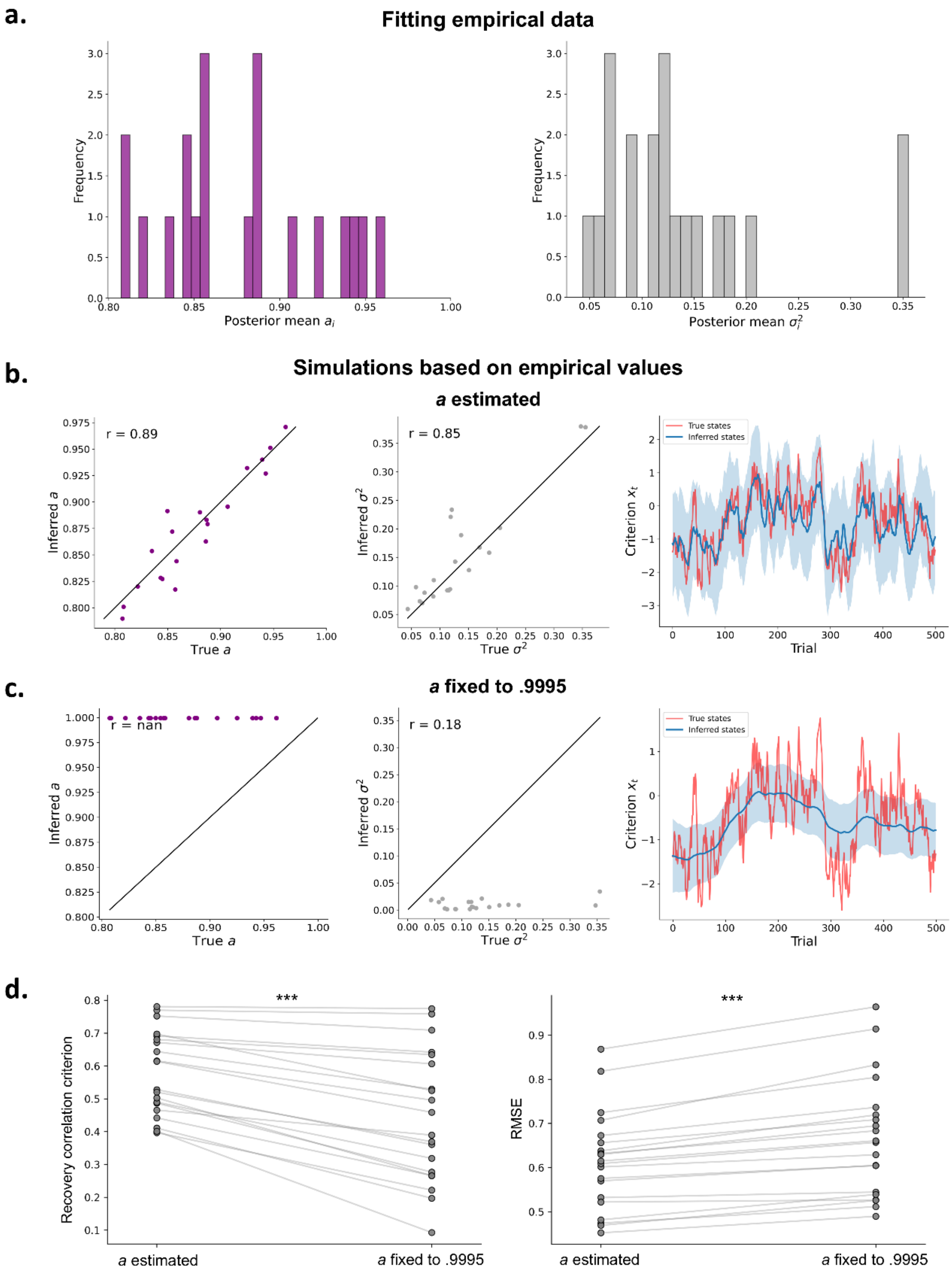

__Figure 7: Applying hMFC to an empirical dataset.__ A) The estimated posterior means for each subject of a_i and σ_i^2 when fitting hMFC to an empirical dataset of Shekhar and Rahnev (2021). Based on these empirical fits of the per-subject parameters we simulated a new dataset and refitted two variants of hMFC where a_i was freely estimated or fixed to .9995 (following Gupta and Brody (2022), Roy et al. (2021)). B) When a_i is freely estimated we see a good recovery for the parameters a_i and σ_i^2, and the criterion trajectory (example subject with first 500 trials shown). C) With a_i fixed to .9995 we see a systematic underestimation of σ_i^2. Most importantly, this also affects the recoverability of the criterion trajectory. D) For all 20 subjects the correlation between the true and estimated criterion trajectory is higher with a_i as a free parameter compared to having it fixed. Similarly, the root mean squared error (RMSE) is lower when a_i is estimated. These results show that it is beneficial to have a_i as a free parameter. However, accurate recovery of a_i at low trial counts requires a hierarchical estimation procedure (Fig. S1).

R1.3 Restructuring required in discussion/demos

The provided code demos seem to be a collection of .py python files (presumably converted from .ipynb Jupyter notebooks) - this format is not interactive, and does not allow other researchers to easily run individual sections, replace with their own datasets or compare their output to reference outputs from the authors' runs. For the sake of reproducibility and ease of use, I suggest that the authors **provide the original Jupyter notebooks (.ipynb) showing the expected outputs of each cell and allowing anyone to easily interact with their demos.** The discussion is currently a bit impoverished, moreover a number of sections currently in the introduction (everything from line 80: **"Why should we care about fluctuations in decision criterion?" onwards until the methods) are more suited to the discussion** as they discuss significance of the authors' methods/results w.r.t previous work, and I would suggest they be moved to the discussion.

We thank the reviewer for these suggestions. We have made the demo available in Jupyter notebooks to improve the accessibility for the user. Regarding the suggestion to move part of our current introduction to the Discussion. We have carefully discussed the pros and cons of this option among the co-authors and eventually have decided to keep the original structure. We think the reader would otherwise not know why it is important to be able to measure criterion fluctuations before reading the methods. However, we have extended the discussion with additional limitations of the model the reader should be aware of on p. 25, lines 464-479 and pasted below:

> *"Although hMFC confers several advantages, it is not without limitations. A first limitation is that it only allows an AR(1) process to govern the decision criterion, i.e., the intercept of the model, whereas it assumes all other parameters are fixed. Future work may explore how to extend this hierarchical Bayesian framework to simultaneously infer fluctuations in the weights of other covariates, e.g. those reflecting stimulus sensitivity or trial history (Roy et al., 2021). A second limitation is that hMFC uses observed choices to estimate latent trajectories, but it does not consider reaction times. Future work may explore how to extend this approach to also incorporate reaction times in the estimation of the latent states of parameters. Finally, as showcased in the paper, hMFC works very well for*

estimating the latent trajectory of the decision criterion. Therefore, researchers seeking to infer those trajectories in their datasets will find hMFC a useful tool. Researchers that are, however, interested in the parameters giving rise to these fluctuations (e.g., a_i and σ_i^2) should carefully inspect the parameter recovery results to determine in which cases they can interpret these estimates. Indeed, for very low trial counts or low number of participants some group-level parameter estimates are not always within the 95% credibility interval of the true values. In those cases, focusing on acquiring data from subjects or more trials per subject could effectively alleviate these concerns."

R1.4 Line 214-217, 235 - 237: Unclear what this is referring to, since the parameterization in Gupta and Brody does not seem to have any parameter weighing the criterion fluctuations

Before developing hMFC we first investigated the model by Gupta and Brody. When running the parameter recovery we noticed a trade-off between σ_i^2 and C, something we also discussed over a meeting with Diksha Gupta. The trade-off is shown in an (older) plot below, with each dot representing one simulated subject and the different panels referring to the number of trials per subject. The parameter C weighs the criterion fluctuations x_t in the Bernoulli probability. Therefore, C effectively acts as a scaling parameter on the criterion fluctuations, with values larger than 1 increasing the scale and values smaller than 1 decreasing the scale. However, σ_i^2 also acts as a scaling parameter. Having both parameters in the same models therefore leads to a trade-off. In hMFC C is not treated as a free parameter and fixed to 1, hereby solving this trade-off. We do agree that this trade-off is not clear from the paper without digging in the code. Since this is only a minor point in the hMFC paper and to avoid further confusing we have therefore decided to simply leave these sentences out of the paper.

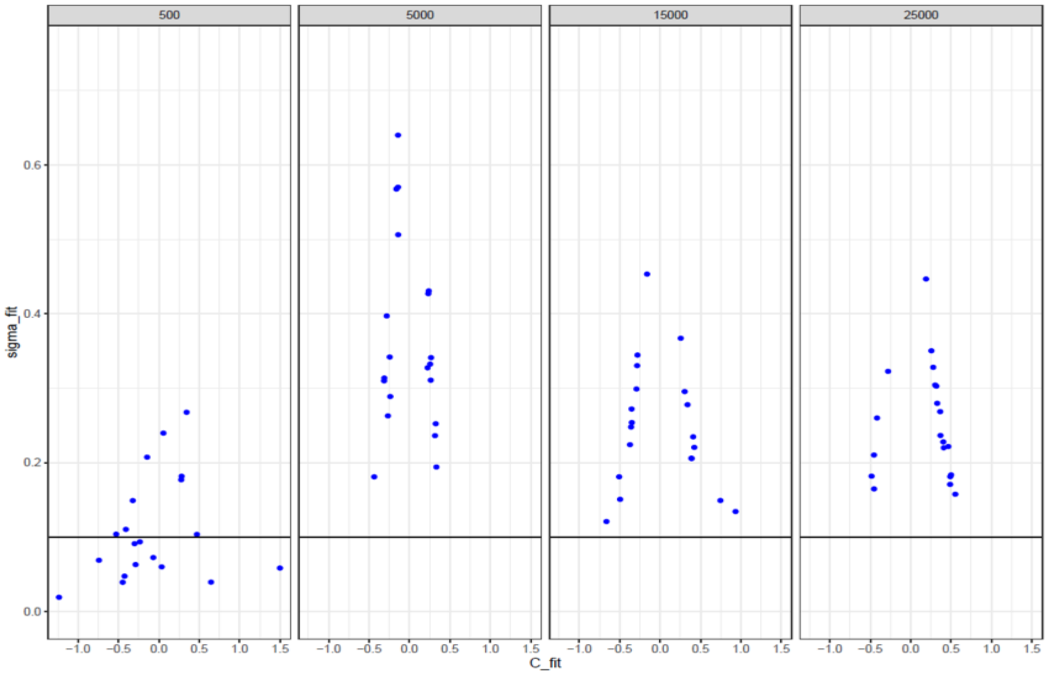

Results from a parameter recovery with the model of Gupta and Brody (2022). The model features a trade-off between the error variance of the AR(1), and the parameter C, which ways the estimated criterion trajectory in the Bernoulli probability. Since both parameters acts a scaling parameter, having both in the same model results in a trade-off.

R1.5 The authors sometime refer to the drifting latent criterion as a “latent state” when describing the model - better to use consistent terminology and refer to the drifting criterion everywhere.

We have updated the manuscript using consistent terminology.

R1.6 Include citation to Dynamax

The Dynamax citation is added in the manuscript.

R1.7 Legends in s3 are not matched to the plotted range

The colors of the legends in S3 are updated.

R1.8 Fig 6 legend criterion estimated v.s. not estimated -> criterion is AR(1) v.s. criterion is fixed

The legend in Figure 6 is updated.

Reviewer #2

In this paper, Vloeberghs and colleagues propose a model (and provide the corresponding Python code on a github repository) for estimating autocorrelated fluctuations in the decision criterion in a two-alternative forced-choice task. The paper begins by presenting the unfortunate consequences of ignoring such fluctuations. When not taken into account, fluctuations in the decision criterion are mistaken for reduced sensitivity (psychometric slope, d') and sequential effects (with an apparently high probability of repeating the previous choice). The paper shows (at the end) that when random drift in the decision criterion is taken into account, the estimates of sequential effects and sensitivity are no longer biased. The proposed model differs from previous models in that it explicitly models the random fluctuations in the criterion (unlike Lak et al 2020), it estimates the temporal dependence of the autocorrelation (unlike Roy et al 2021), and it uses a hierarchical fitting approach to regularise the estimates in the absence of a large number of trials (unlike what is done e.g. with rodents, Gupta & Brody 22). I have looked at (but not tested) the code. It appears to be well written and documented. It also seems to be fast because it is written using jax, which makes it possible to benefit from faster computation time when GPUs are available, without the user having to adapt the code. The appendix explains the mathematics implemented in the code very clearly, it's easy to follow.

Overall, I think this methodological/software paper is useful and very well written. It draws the community's attention to the problem of variability in the decision criterion and its consequences, and proposes a solution that is applicable in typical human experiments (where the number of trials is typically limited) and is readily available in Python in a nicely documented repository. The paper presents a number of quality checks for their methods and code.

R2.1 I have only one request regarding the parameter recovery presented in Figure 3 and Figure 4. One aspect is missing: we would also like to know to what extent the parameters can be distinguished from each other by **reporting the correlations between the fitted values of parameter k and the true value of parameter j** (and not only the special case j=k presented in Figure 4). This is particularly interesting for parameters a and \sigma of the autocorrelation model.

We thank the reviewer for this suggestion. Below we have included a plot that shows the correlations for the subject-level parameters for one representative dataset with 50 subjects with 500 trials each. Each dot represents one subject. Apart from the diagonal, no systematic correlations across all datasets are present, suggesting that our model is perfectly able to distinguish all subject-level parameters. We now refer to this Figure in the Manuscript on p. 19, lines 384-386:

> *"The autocorrelation of the posterior samples looks good (Fig. S6) and no systematic correlations were present across the true and inferred values of different parameters (Fig. S7)."*

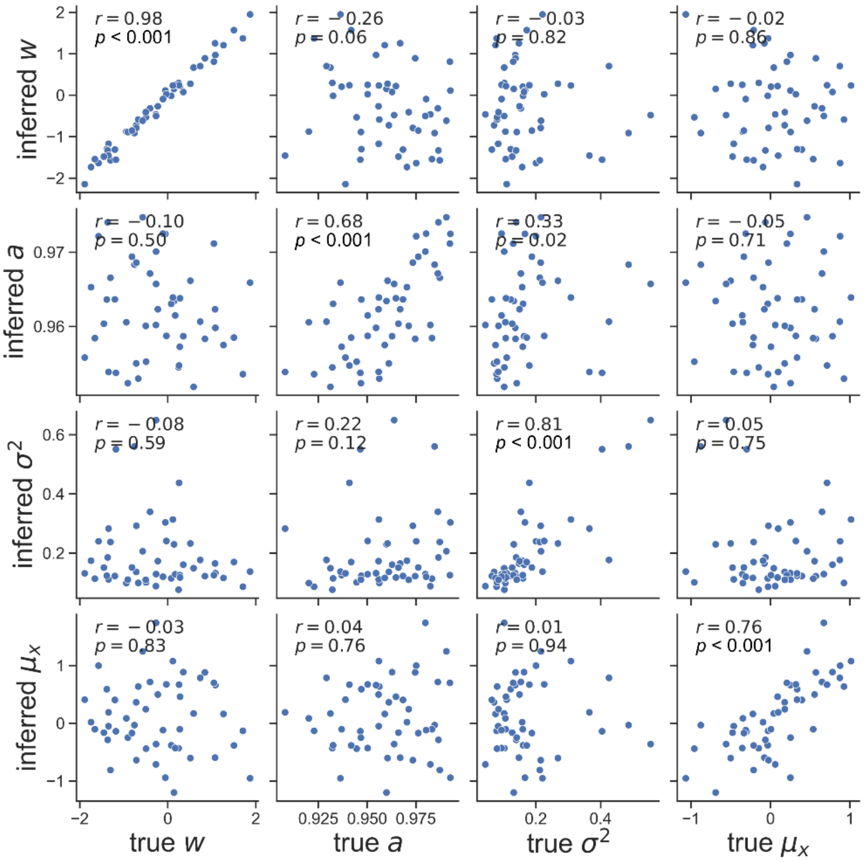

Supplementary Figure S7: The correlations between the true and inferred per-subject parameters for one example dataset (50 subject with 500 trials each). Apart from the diagonal there were no systematic correlations present across the datasets, indicating that there are no trade-offs between the parameters during estimation.

The above shows that at the subject-level there is a good recovery of parameters, without any trade-off between them. At the group-level, the true value of the parameters is the same for all simulated datasets, hence assessing the correlation between true and estimated values over datasets is not possible. Nevertheless, we did investigate the correlation between the inferred values of the different parameters and discovered a very high correlation (r = .95, p < .001) between the inferred values for α_{σ^2} and β_{σ^2}, the parameters of the hierarchical prior for σ^2. This is presumably due to the mean of the inverse gamma (IG) distribution being $\mu_{\sigma^2} = \beta_{\sigma^2} / (\alpha_{\sigma^2}-1)$. Consequently, the same value for μ_{σ^2} can be obtained with different combinations of α_{σ^2} and β_{σ^2}. Such a high and almost perfect correlation between parameters could jeopardize proper mixing of the Gibbs samples, resulting in high autocorrelation in the traces and a reduced effective sample size. We therefore changed the model structure and reparametrized this hierarchical prior. Instead of estimating α_{σ^2} and β_{σ^2} we now estimate μ_{σ^2} and β_{σ^2}. With these estimates α_{σ^2} can then calculated as $\beta_{\sigma^2} / \mu_{\sigma^2} + 1$. In Supplementary Figure S5 with this reparameterization the correlation between μ_{σ^2} and β_{σ^2} is .12 (p = .39), hereby successfully removing the correlation between the two parameters of the IG prior. It should be noted that μ_a and σ_a are significantly correlated (r = 0.53, p < .001). However, this does not pose a problem for the model. First, there is an excellent recovery for both parameters, with the true value being within the 95% credible

interval for at least 46 out of 50 datasets, even with small numbers of subject or trials (see Figure S3). Second, as mentioned earlier, high correlations can result in high autocorrelation and a lower effective sample size. However, this is not the case as the autocorrelation for both parameter rapidly declines over lags (see Figure S7). Lastly, we do not expect these parameters to be uncorrelated under the posterior, so it is appropriate that the model can recover it when a correlation exists. We now refer to this Figure in the Manuscript on p. 19, lines 373-374:

"No problematic correlations between the inferred values of different parameters were observed, suggesting that the model can distinguish all parameters (Figure S5)."

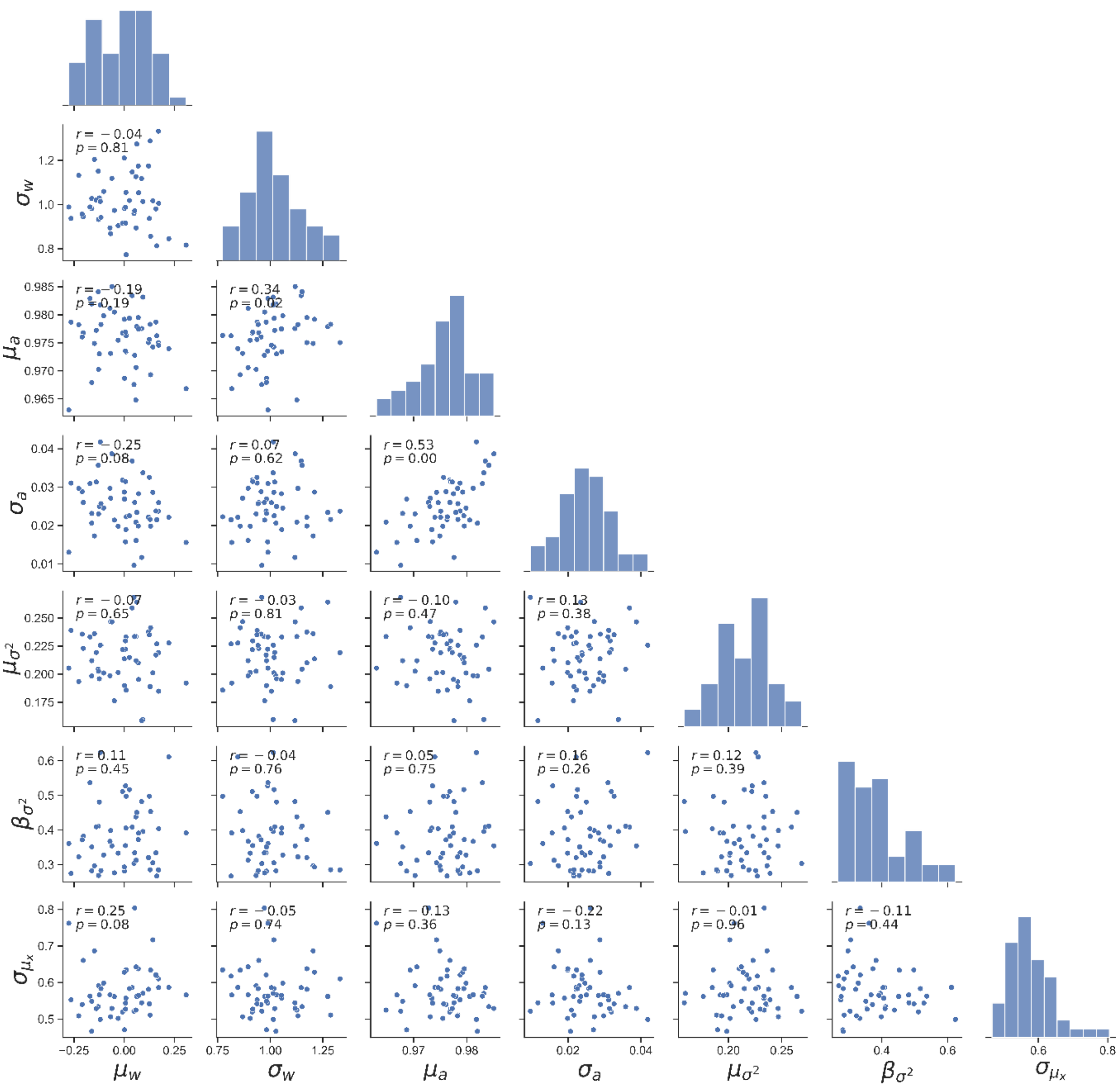

Figure S5: The correlations between the inferred values of the group-level parameters across 50 simulated dataset with 50 subjects per dataset and 500 trials per subject.

Reviewer #3:

This paper introduces a hierarchical Bayesian model for estimating parameters that capture fluctuations in the decision criterion. The model effectively estimates these parameters at both individual and population levels, even with limited data. The authors demonstrate that neglecting fluctuating decision criteria can lead to misinterpretations, such as attributing data to choice history biases or underestimating perceptual sensitivity. With growing recognition of the importance of understanding and accounting for fluctuations in decision criteria, the ability to do so with fewer trials makes this approach highly appealing to a broader community of researchers interested in both behavioral and neural mechanisms of decision-making.

The paper is very well written with detailed explanations of the potential mis-interpretations that can happen when fluctuating criterion is not accounted for. The results are rigorous and figures are easy to understand. However, I do have a few small questions:

R3.1 The authors claim that not accounting for decision fluctuations can lead to misinterpretations of the data (fig 1c-e). However, my concern is, if a subject does indeed have choice history bias, fitting this model might incorrectly attribute that to fluctuating decision criterion. **Can this model be extended to account for both?** Or it is for researchers using this tool to decide if they want to fit two separate models and do model comparison to know the true underlying mechanism.

We thank the reviewer for this valid concern. The model in its current form can effectively account for both. If a subject has a choice history bias driven by systematic updating in function of the previous response (i.e., slope ≠ 0), this will be captured by hMFC through the slope w for previous response and not be attributed to the criterion fluctuations. To demonstrate that the model can correctly disentangle these two sources of choice history bias we re-simulated Figure 6 but now in addition to the criterion fluctuations we also included a systematic effect of previous response. Therefore, the observed choice history bias is a combination of criterion fluctuations and a previous response effect. In Figure S9 below when there are no criterion fluctuations (a = 0, σ^2 = 0) the systematic updating in function of previous response results in a choice history bias with P(repeat response) higher than 0.5. When criterion fluctuations are present we see that this repetition effect further increases.

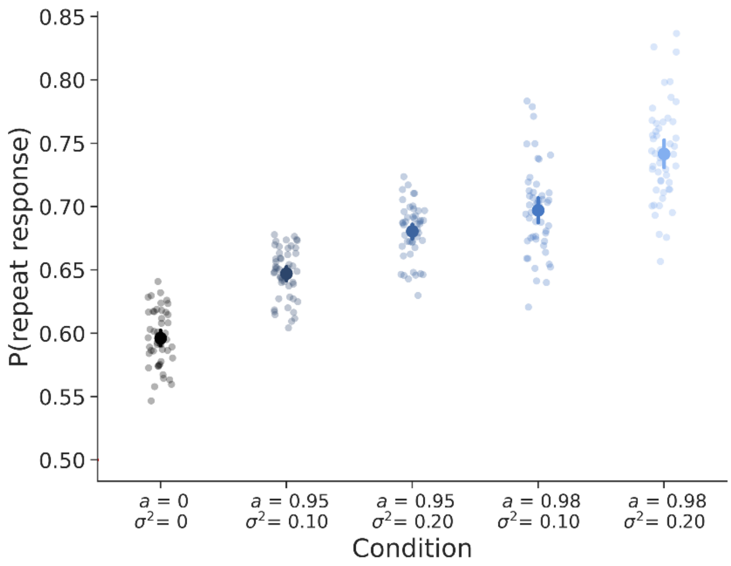

Figure S9: The probability of repeating the previous response for 50 simulated subjects with 5000 trials each, for different conditions of criterion fluctuations. In contrast to Figure 1C, there is now systematic updating in function of the previous response ($w_{\text{previous response}}$ = 0.50), resulting in a P(repeat response) ≠ 0.50 with a stable criterion (a = 0, σ^2 = 0).

Next we fitted hMFC to these simulated data. The figure below shows the posterior distributions for the slope w of previous response for the different simulation conditions. When hMFC estimates criterion fluctuations we see that the true value for the previous response slope (w = 0.5) falls in the posterior distribution for all conditions, even when no true criterion fluctuations were present in the data (Figure 6). However, when the fluctuations are present but hMFC does not estimate them (i.e., assumes a fixed criterion) the fluctuations are absorbed by the previous response slope, resulting in an inflated estimate. These results show that not estimating criterion fluctuations while they are present leads to an apparent (or inflated) repetition effect. Most importantly, it also shows that hMFC can correctly distinguish between the two sources of choice history bias, namely a systematic effect of previous response and criterion fluctuations. So if a researcher is interested in the true mechanism underlying choice history bias, they directly can use hMFC without any adjustments. We now explain this in more detail in the Manuscript on p. 22, lines 408-426:

> *"We fit the hMFC model with stimulus evidence and previous response as covariates, similar to the data shown in Figure 1 but now with $w_{\text{previous response}}$ = 0.5. As a consequence, there is a repetition effect even when no criterion fluctuations are present (Figure S9). As the criterion fluctuations become stronger, this repetition effect increases. Importantly, we ran the fits either with or without estimating criterion fluctuations, allowing us to test if the model can accurately distinguish between these two sources underlying the repetition effect.*
>
> *When hMFC estimates criterion fluctuations, the true value of the previous response slope ($w_{\text{previous response}}$= 0.5, red line) falls within the posterior distribution across all conditions—even when the data contain no actual criterion fluctuations (Figure 6A). However, when the fluctuations are present but hMFC does not estimate them (i.e.,*

assumes a fixed criterion) the repetition effects driven by the criterion fluctuations are absorbed by the previous response slope, resulting in an inflated estimate for $w_{\text{previous response}}$. *As the criterion fluctuations become stronger the posterior distributions are shifted further away from the true value (i.e., replicating the finding from Figure 1C, using our model fit). These results show that not estimating criterion fluctuations while they are present leads to inflated estimates for the systematic effect of previous response. Most importantly, it also shows that hMFC can correctly distinguish between the two sources of choice history bias, namely a systematic effect of previous response and criterion fluctuations."*

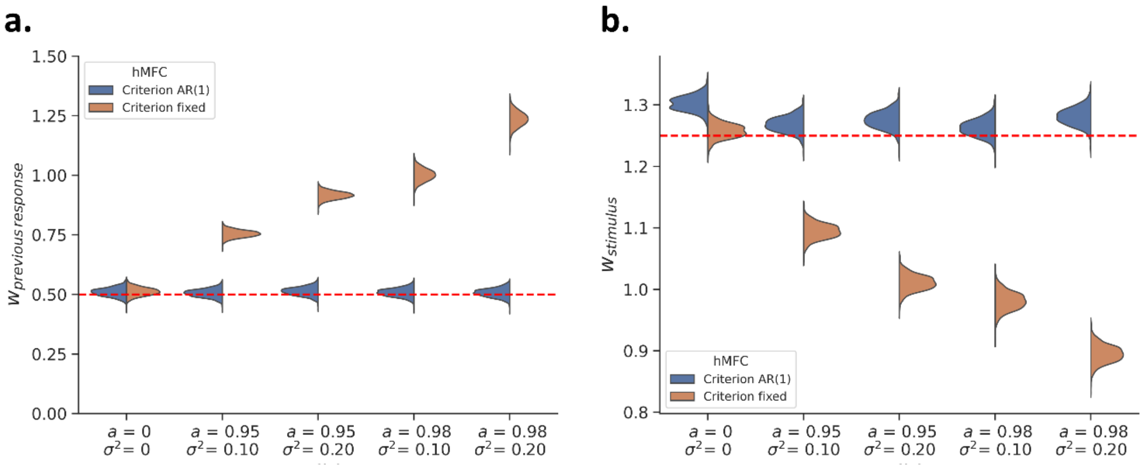

Figure 6: The recovered posterior distributions with the criterion estimated as AR(1) or assumed to be fixed (i.e., no fluctuations are estimated). When criterion fluctuations are present but not estimated the posterior estimates for $w_{previous\ response}$ *are inflated (A) whereas for* $w_{stimulus}$ *they are underestimated (B). In contrast, when the criterion fluctuations are accounted for with hMFC we see a correct recovery of the posterior estimates for both parameters. Estimating criterion fluctuations with hMFC therefore helps to address biases in these parameters.*

R3.2 Additionally, it would add value to see if the authors can use some of the available **empirical data** in literature to fit this model and explain the observations.

To show the benefits of estimating a_i *as a free parameter, we now showcase the results of fitting hMFC to the empirical dataset of Shekhar & Rahnev (2021) consisting of 20 subjects with each 2790 trials. As can be seen in the figure below, hMFC estimates for* a_i *values between 0.81 and 0.96, and* σ_i^2 *values between 0.05 and 0.35. In addition, the model recovers the latent state of the criterion fluctuations for all of these participants. In panel C two example subjects are shown. These fits demonstrate that hMFC effectively estimates* a_i, σ_i^2 *and latent states from empirical data. Panels A and B are added to the main text in in Figure 7.*

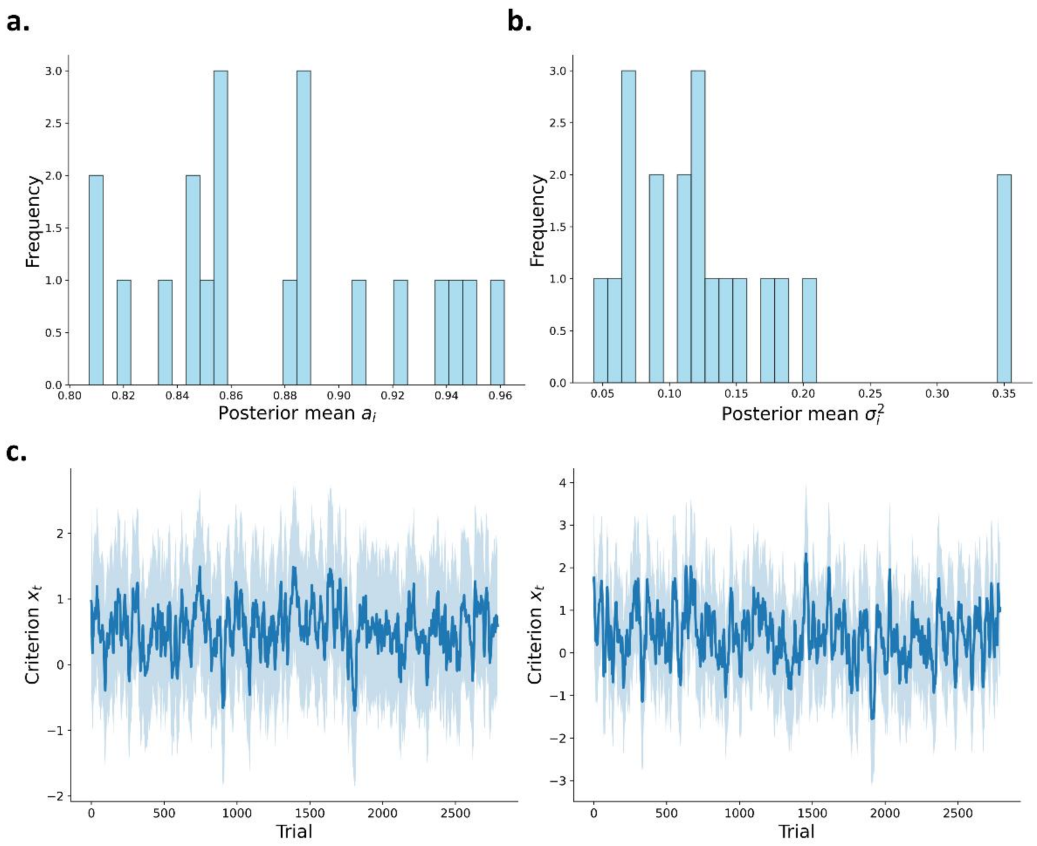

Fitting hMFC to empirical data of Shekhar and Rahnev (2021). Estimates for the per-subject a_i (A) and σ_i^2 (B). C) Criterion fluctuations of two example subjects.

R3.3 Since the authors are using Gibbs sampling, I was wondering if they could highlight some **parameter ranges where the algorithm would not be able to converge with limited iterations due to correlation of variables**

To address this question we ran additional simulations (50 subjects with 500 trial each) where we manipulated the precision σ_w of the normal hierarchical priors for w_i. With different levels of σ_w for the different input variables we effectively can make the per-subject w_i correlated with each other. That is, given the group-level mean μ_w they are conditionally independent but marginally they are dependent. As can be seen below, the samples of μ_w mix more slowly when σ_w becomes smaller.

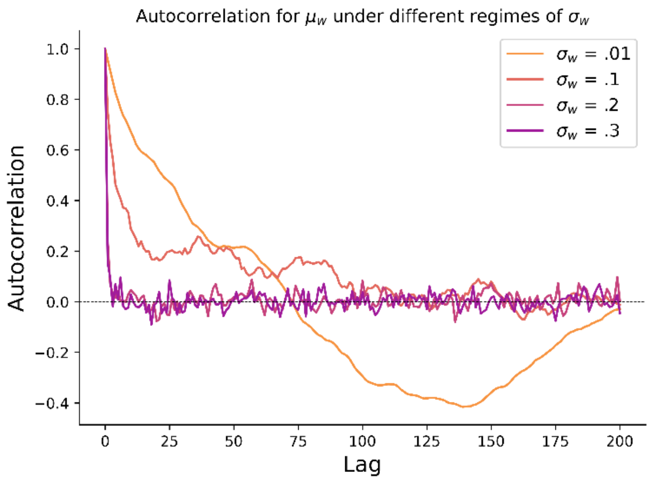

The autocorrelation of the posterior samples for μ_w for different levels of σ_w. Posterior samples show a higher autocorrelation with a smaller σ_w.

Next, we looked at the autocorrelations of the group-level and per-subject parameters for the full model from the parameter recovery reported in the paper (Figure 3-5). In Figure S7 a representative dataset (50 subjects, 500 trials) is shown. First, for the group-level parameter we see that the autocorrelation quickly decays over lags (panel A). Only for β_{σ^2} it takes slightly longer compared to the other parameters. As this indicates slower mixing of the posterior values, more samples and fitting iterations and are warranted. For the per-subject parameters the autocorrelation quickly drops except for σ_i^2. Presumably, this is related to the longer autocorrelation of it hierarchical priors β_{σ^2}.

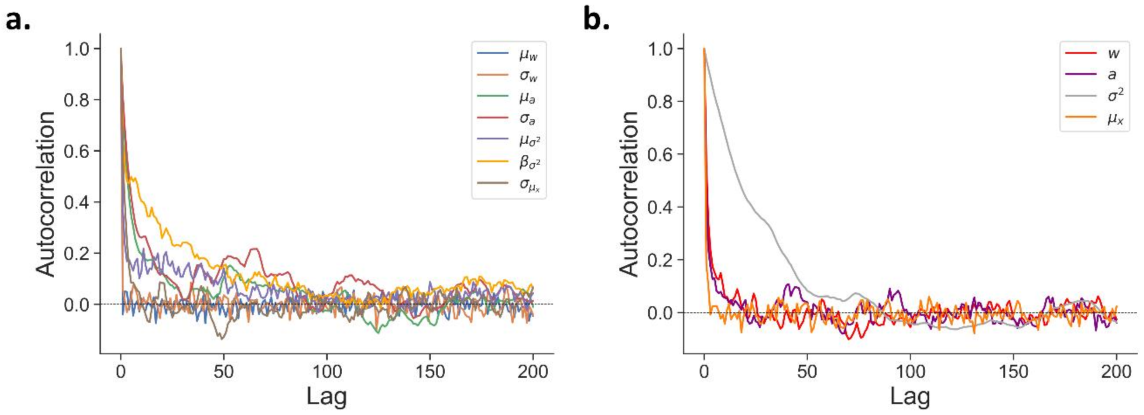

Figure S6: The autocorrelation for the group-level parameters (A) and per-subject level parameters (B) for one representative dataset of 50 subjects with 500 trials each.

We now refer to this Figure in the Manuscript on p.19, lines 373-376 and lines 384-386:

“No problematic correlations between the inferred values of different parameters were observed, suggesting that the model can distinguish all parameters (Figure S5). Lastly, the autocorrelation of the posterior samples quickly decays over lags, suggesting good mixing of the samples (Figure S6).”

“The autocorrelation of the posterior samples looks good (fig. S6) and no systematic correlations were present across the true and inferred values of different parameters (fig. S7).”

R3.4 Finally, it would help to discuss some **limitations of the model/tool**, maybe the top few issues that researchers using the tool should be aware of.

As requested, we addressed some limitations of hMFC in the discussion on p. 25, lines 464-479.

> *“Although hMFC confers several advantages, it is not without limitations. A first limitation is that it only allows an AR(1) process to govern the decision criterion, i.e., the intercept of the model, whereas it assumes all other parameters are fixed. Future work may explore how to extend this hierarchical Bayesian framework to simultaneously infer fluctuations in the weights of other covariates, e.g. those reflecting stimulus sensitivity or trial history (Roy et al., 2021). A second limitation is that hMFC uses observed choices to estimate latent trajectories, but it does not consider reaction times. Future work may explore how to extend this approach to also incorporate reaction times in the estimation of the latent states of parameters. Finally, as showcased in the paper, hMFC works very well for estimating the latent trajectory of the decision criterion. Therefore, researchers seeking to infer those trajectories in their datasets will find hMFC a useful tool. Researchers that are, however, interested in the parameters giving rise to these fluctuations (e.g., a_i and σ_i^2) should carefully inspect the parameter recovery results to determine in which cases they can interpret these estimates. Indeed, for very low trial counts or low number of participants some group-level parameter estimates are not always within the 95% credibility interval of the true values. In those cases, focusing on acquiring data from subjects or more trials per subject could effectively alleviate these concerns.”*
